# Biological Activity of Canned Pork Meat Fortified Black Currant Leaf Extract: In Vitro, In Silico, and Molecular Docking Study

**DOI:** 10.3390/molecules28248009

**Published:** 2023-12-08

**Authors:** Karolina M. Wójciak, Paulina Kęska

**Affiliations:** Department of Animal Food Technology, Faculty of Food Science and Biotechnology, University of Life Sciences in Lublin, Skromna 8, 20-704 Lublin, Poland; karolina.wojciak@up.lublin.pl

**Keywords:** canned meat, functional food, bioactivity, bioinformatics analysis

## Abstract

The aim of this study was to assess the antioxidant and inhibiting (ACE-I, DPP IV, and alpha-glucosidase) potential of canned meat featuring reduced sodium nitrate content (50 mg/kg) and fortified with freeze-dried currant leaf extract. Research indicates that employing a lyophilizate dose of 150 mg/kg yields optimal benefits in terms of the antioxidant activity of the meat product. Additionally, three highly promising sequences for canned meat were identified via analysis in the BIOPEP database. These sequences are RPPPPPPPPAD, exhibiting DPP-IV inhibiting activity; ARPPPGPPPLGPPPPGP, demonstrating ACE-I inhibiting activity; and PPGPPPPP, displaying alpha-glucosidase inhibiting activity. Using bioinformatics tools, molecular docking was performed by pairing the selected peptides with protein receptors 2QT9, 1O86, and 5NN8, respectively (PDB ID). The examination of the potential of these selected sequences to manifest specific biological activities toward enzymes was based on the free energy value (∆Gbinding). This knowledge can be harnessed for designing functional foods, thereby contributing to the safeguarding of consumer health.

## 1. Introduction

Meat and meat products serve as a crucial source of essential nutrients, including protein, fatty acids, vitamins, minerals, and other bioactive compounds. Consequently, they should constitute a foundational element in a well-rounded diet. Nevertheless, processed meat products also incorporate ingredients suspected to elevate the risk of specific cancers when consumed inadequately, such as sodium nitrates. Concurrently, the increasing comprehension of the correlation between diet and health among consumers, coupled with their interest in foods with additional health-promoting functions, contributes to the evolution of functional foods. Emerging products within the meat industry, aligning with the definition of functional food, should facilitate meeting consumer needs and align with prevailing recommendations on nutritional and dietary goals [1,2]. The dietary patterns of consumers whose primary food intake consists of animal products are deeply ingrained. It appears improbable that they would entirely forgo meat consumption. However, to align with evolving food trends and dietary guidelines, there is potential to modify meat products by incorporating nonmeat ingredients acceptable to consumers. One such example is the integration of plants, which serve as excellent carriers of bioactive compounds in the diet. The utilization of plant additives is primarily associated with enriching the product with diverse bioactive ingredients, resulting in specific meat-based products with attributes that confer health-promoting properties [3,4]. Extracts from plant materials, including fruits, vegetables, herbs, spices, and their components, represent a valuable source of natural antioxidants [5,6]. Notably, phenolic compounds, a significant component of natural antioxidants from plants, have garnered substantial attention for their impressive radical scavenging activity and reducing capacity.

Fruit and leafy herb extracts underwent testing for their antioxidant activity and their ability to inhibit oxidative degradation in meat products. Polyphenols play a role in inhibiting oxidative processes by (a) acting as scavengers for reactive species, (b) inhibiting lipoxygenase, and (c) reducing metmyoglobin. Consequently, polyphenols emerge as potential antioxidants for meat and meat products [7]. For instance, McCarthy et al. [8] conducted an assessment of the antioxidant effects of aloe, fenugreek, ginseng, mustard, rosemary, sage, and tea catechins in pork patties. The researchers noted that the catechins present in tea, rosemary, and sage extracts exhibited potent antioxidant properties. Similarly, Nissen et al. [9] gauged the antioxidant effectiveness of select plant extracts, ranking their antioxidant activity as follows (in descending order): rosemary > grape skin > tea > coffee. Hassan and Fan [10] underscored the benefits of green tea extract (200 mg/kg) and cocoa leaf extract (800 mg/kg), showcasing antioxidant properties akin to the BHA/BHT blend. Curry leaf and mint extracts also demonstrated potential for use as natural antioxidants in pork products, with curry leaf extract displaying more favorable effects [11]. Moreover, Nowak et al. [12] highlighted the potential of cherry and blackcurrant leaves as effective natural antioxidants, safeguarding against lipid oxidation in pork meat products during refrigerated storage. These instances of employing plant extracts to enhance the quality of meat and meat products, inhibiting pro-oxidative changes, primarily focused on lipids within the product. Notably, the oxidation of fatty acids stands out as a major contributor to quality deterioration, leading to color and texture degradation, off flavors, and odor (rancidity), along with nutrient loss. The rancidity of fats is linked to the formation of secondary oxidation products, posing health risks as markers of oxidative stress associated with accelerated aging of body cells, contributing to an increased incidence of non-communicable diseases. Lipid and protein oxidation involve intricate mechanisms and reaction processes that are interdependent; an intensification of fat oxidation strongly promotes increased protein oxidation. Both of these oxidative processes primarily occur via a radical chain reaction involving initiation, propagation, and termination stages [13,14]. Consequently, an effective approach to slowing down oxidation involves using antioxidants to disrupt the radical chain reaction, preserving the nutritional and sensory properties of meat products. Previously, we demonstrated the feasibility of producing canned meat fortified with lyophilized extract of black currant leaves, incorporating a reduced content of sodium nitrates. The utilized extracts contained significant amounts of phenolic acids and flavonoid derivatives (the chemical content of extracts was presented in [15]), contributing to their antioxidant activity and their ability to inhibit the growth of selected Gram-positive bacteria. This resulted in canned products where the addition of 150 mg/kg of blackcurrant leaf extract led to a reduction in oxidative transformations of fat in meat products during 180 days of storage without adversely affecting color parameters and health safety (products of good microbiological quality and without *N*-nitrosamines) [15]. Therefore, the use of these plant additives, containing antioxidant compounds, is viable without compromising the quality of meat products with reduced nitrates. Nevertheless, much remains unknown regarding the impact of this treatment on the overall biofunctionality of the product. Meat contains various bioactive compounds, for example protein-derived compounds like peptides. These compounds can have multifunctional effects, potentially enhancing consumer health, at least in vitro. They may act as preventive factors or support the treatment of diet-related noncommunicable diseases, such as hypertension, obesity, diabetes, and others [16,17]. However, it is crucial to consider the effect of using plant additives on the action of these biomolecules. Polyphenols are recognized for exhibiting binding affinity to proteins via noncovalent and/or covalent interactions, offering a strategy for engineering polyphenol-protein complexes to protect them from oxidation and enzymatic hydrolysis during digestion in the gastrointestinal tract [18]. As highlighted by Li et al. [19], the interaction between polyphenols and proteins alters the physical and chemical properties of polyphenols, shielding them from oxidation and enzymatic hydrolysis during digestion in the gastrointestinal tract, thereby improving their absorption rate, delivery to the target, and biological activity. Polyphenols in complex with proteins may also provide a protective effect on proteins in the meat product, safeguarding them against oxidative factors or protecting them from exposure to endoproteases, especially during prolonged storage. Thus, the peptide profile after 180 days of storage and their bioactivity may hinge on the type, dose, and storage period of canned pork fortified with black currant leaf extract.

In this study, we aim to validate the hypothesis regarding the protective impact of blackcurrant leaf extracts on alterations in the biological properties of proteins and peptides released from canned meat. To achieve this objective, we isolated, identified, and then subjected the peptides’ sequences isolated from canned meat after 180 days of refrigerated (4 °C) storage to in silico analysis. For the peptides showing the most promise, we conducted molecular docking analyses to assess their potential antihypertensive (as angiotensin I converting enzyme (ACE-I)) and antidiabetic (as dipeptidyl peptidase IV (DPP-IV) and alpha-glycosidase inhibitors) effects. Furthermore, we evaluated the in vitro antioxidant activity of peptide isolates from canned meat using ABTS, chelate iron (II) ions, and Reducing Power assays. Selected assays were also carried out immediately after production (day 1) to facilitate a more comprehensive discussion of the results.

## 2. Results and Discussion

### 2.1. Spectrometric Characteristic of Peptides Isolated from Canned Meat with Black Currant Leaf Extract

**Figure 1:**
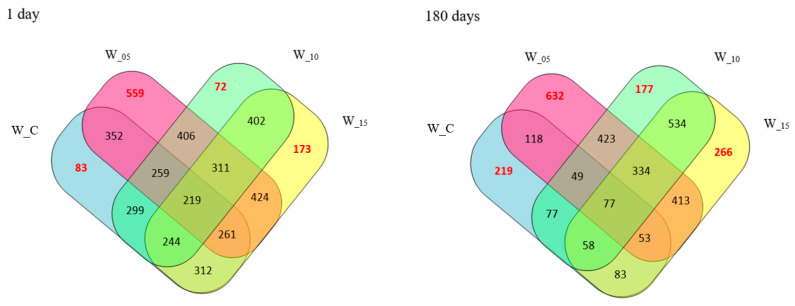
Sequence similarity of the peptides obtained in canned pork meat (numbers indicate the number of identical sequences determined between the designated samples) (W_C—control; W_05—black currant leaves extract 50 mg/kg; W_10—black currant leaves extract 100 mg/kg; W_15—black currant leaves extract 150 mg/kg).

The acquired peptides underwent analysis using mass spectrometry to determine both the molecular masses and the sequence of the peptides. In total, around 2000 peptide sequences were identified, appearing either individually (characterizing a specific sample) or concurrently in multiple samples (common) across various assay variants (Table 1). Elaborate quantitative attributes of the peptides for each analyzed variant, along with their interrelationships, are depicted via a Venn diagram (Figure 1).

**Figure 2:**
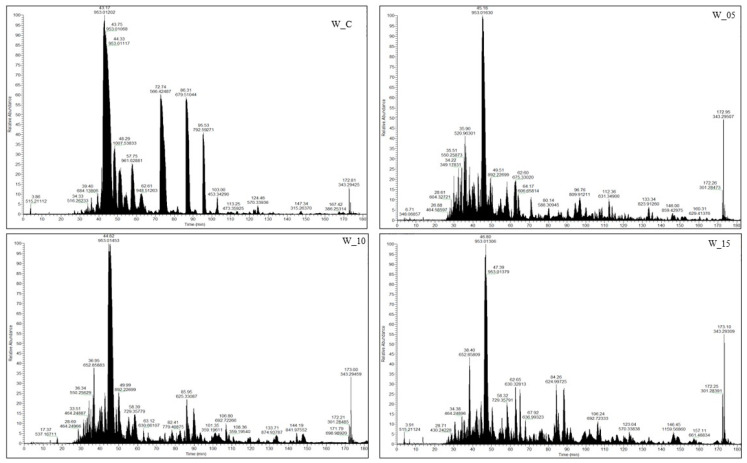
Representative base peak chromatogram spectra peptides from canned meat products after 180 days of storage (W_C—control; W_05—black currant leaves extract 50 mg/kg; W_10—black currant leaves extract 100 mg/kg; W_15—black currant leaves extract 150 mg/kg).

Among all the research samples, the control samples (W_C) exhibited the lowest peptide content, aligning with findings from a comparable study on the effects of willow herb extracts by Ferysiuk et al. [20]. The impact of adding freeze-dried currant leaf extract on the total peptide content was also observed. This content decreased with the increasing dose of the plant additive in samples featuring a reduced content of sodium nitrate (III). Notably, the peptide content in sample W_15 (with the dose of sodium nitrate (III) halved and the highest dose of lyophilizate) closely resembled that of W_C (with the addition of sodium nitrate (III) at the level of 100 mg/kg), both immediately after production (day 0) and following storage (180 days). Consequently, these results further validate the influence of lyophilizate addition and its dose on the peptide profile. Additionally, the impact of storage time on the peptide content identified via the spectrometric method was observed, and the results are presented in Figure 2. A decrease in the number of peptides across all analyzed samples and an increase in sequences characteristic of the variant were noted after 180 days of storage. Notably, research samples subjected to additional technological treatment (W_05, W_10, W_15) differed from the control samples (W_C). The distinctions in the peptide profile between the variants are also affirmed by the imaging of the spectrophotometric analysis (Figure 2).

Following 180 days of storage, a reduction in peptide content was observed in the control sample (W_C), while an increase was noted in samples with the addition of lyophilized aqueous extract of currant leaves and a reduced dose (50 mg/kg) of sodium nitrate III (W_05). A 26% and 40% increase in peptide content was recorded in samples W_10 and W_05, respectively. The diverse trends observed in this study may result from biochemical processes occurring with varying intensity in the product. These processes could be influenced, for example, by interactions between canned ingredients and plant additives (such as the interaction of peptides with phytochemicals) or different levels of protection against oxidative factors (caused, for instance, by varying doses of sodium nitrate (III)). Polyphenols have the potential to reduce the intensity of peroxidation of primary and secondary lipids, restrict the formation of reactive hydroperoxides or epoxides by inhibiting lipoxygenase activity, minimize the degradation of salt-soluble myofibrillar proteins and sulfhydryl groups, and delay the growth of bacteria [7]. Studies in molecular and muscle models have demonstrated that polyphenols and polyphenol-rich extracts can stabilize the color of meat products by inhibiting myoglobin oxidation, reducing the amount of metmyoglobin, or both. Inai et al. [21] investigated the ability of polyphenols to reduce metmyoglobin to oxymyoglobin forms, showing a robust effect of polyphenols on metmyoglobin. Primarily, flavonols (kaempferol, myricetin, and quercetin), along with sinapic acid, catechin, nordihydroguaretic acid, taxifolin, morin, and ferulic acid, were identified as compounds reducing metmyoglobin levels. However, some studies indicate that these phenolic compounds can bind to meat proteins and generate protein crosslinks [22,23]. Plant polyphenols used as natural antioxidant components are known to bind to cysteine residues in meat proteins [24], affecting the structural and functional characteristics of proteins.

### 2.2. Antioxidant Properties of Peptides: In Vitro Analysis

The influence of the dosage of freeze-dried water extract from blackcurrant leaves and the duration of cold storage on the antioxidant activity of peptides, as measured in extracts obtained from sterilized cans, was validated (Table 2).

A correlation was evident between the content (dose) of freeze-dried plant extract additive and the antiradical activity of the analyzed samples, exhibiting a linear increase according to the following order: W_C < W_05 < W_10 < W_15 (excluding the W_10 sample immediately after production, which recorded the lowest value of antiradical properties measured with the ABTS test among the samples with a plant additive). The antiradical activity values immediately after production ranged from 23.13% for the control sample to 50.97% for the sample with the highest dose of freeze-dried currant leaf extract (W_15). After 180 days of storage, there was an increase in antiradical activity against ABTS•+, with the highest increase recorded for W_10 and the smallest for the control sample (W_C). The study also employed the iron ion chelating capacity of peptide extracts as a method to assess their antioxidant properties. The activity was determined at an average level of 18% after production (day 1) and 24% (except for sample W_15) after a six-month storage period (day 180). After the storage period (180 days), the peptides’ ability to chelate iron ions increased (compared to day 1), irrespective of the analyzed research variant. The lyophilizate dose effect was evident in the results, notably noticeable in tests W_10 and W_15 compared to the control sample (W_C). The lowest values determining the extracts’ ability to chelate iron ions were recorded for the sample without the addition of lyophilizate (W_C). The antioxidant properties of peptides in extracts from canned food were also assessed using the reduction power test, with values presented as the absorbance value measured at 700 nm. A higher value indicates greater reducing properties of the test material. The influence of the lyophilized extract dose (reduction in reducing power with an increasing dose of freeze-dried extract from currant leaves) and the duration of cold storage (increase in reducing power in all analyzed variants) on the discussed parameter was demonstrated. Immediately after production, the control sample (W_C), containing a doubled dose of sodium nitrate (100 mg/kg) compared to the other variants (50 mg/kg), exhibited the highest value of the reduction force. Considering batches with the plant additive, the best reducing properties were found in the trial with the lowest level of lyophilizate addition (W_05) (Table 2). The trends observed in this study align with previous literature reports, indicating that the addition of *E. angustifolium* L. extracts positively impacts the peptide profile and antioxidant activities, with results depending on the amount of extract added to the meat product [20].

**Figure 3:**
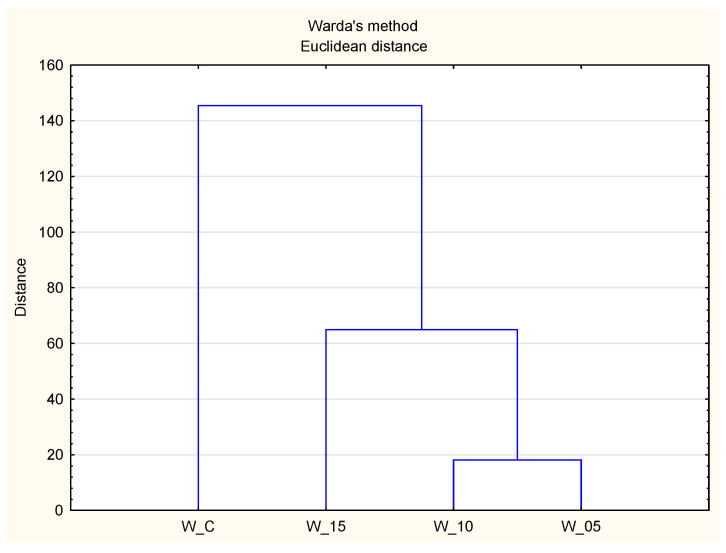
Graphical presentation (HCA dendrogram) of the relationship between samples based on their antioxidant properties (W_C—control; W_05—black currant leaves extract 50 mg/kg; W_10—black currant leaves extract 100 mg/kg; W_15—black currant leaves extract 150 mg/kg).

The results of this study establish the antioxidant activity of peptides derived from canned meat as the fundamental criterion for hierarchical cluster analysis. This method aims to discern relatively uniform groups based on selected properties, utilizing an algorithm that initiates with each variant designated for analysis in a distinct cluster and amalgamates clusters until only one remains. Figure 3 illustrates a dendrogram, providing a graphical representation of the acquired outcomes. The analysis reveals the impact of incorporating freeze-dried currant leaf extract on the antioxidant properties of the meat product. Trials incorporating the extract (W:05, W_10, W_15) form a distinct cluster I, while control samples constitute cluster II. Within cluster I, trials with the highest lyophilizate dose (W_15) exhibit the most distinctive characteristics compared to other variants (W_10, W_05), forming a separate subcluster. Simultaneously, it aligns closely with the cluster represented by W_C. Considering the trends in the quantitative assessment of peptides via spectrometric methods, it is anticipated that the antioxidant activity of meat products is significantly influenced by the peptides within them, further supported by bioactive compounds of plant origin from black currant leaf extract [15].

Due to the highly encouraging findings observed in the canned meat sample with the inclusion of 150 mg/kg of freeze-dried currant leaf extract and a 50% reduction in sodium nitrate levels, this specific variant was chosen for further scrutiny. A thorough analysis will be conducted by comparing it with the outcomes obtained from samples featuring the standard dose of sodium nitrate. 

### 2.3. Bioactive Properties of Peptides from W_C and W_15: In Silico Analysis

**Figure 4:**
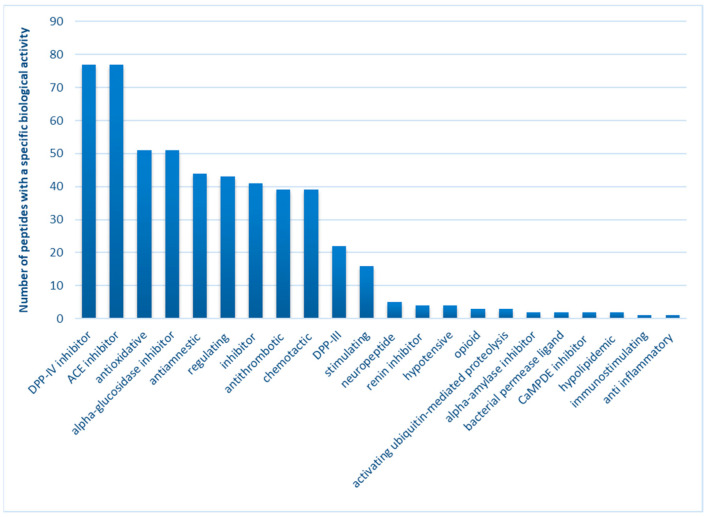
Biological activity profile of peptides obtained from canned meat products.

Considering the latest literature reports, researchers are currently focused on evaluating the biological effects of bioactive compounds, specifically peptides derived from various food sources. This emphasis is placed on assessing their activity against oxidizing agents, a crucial aspect not only for the quality of food products dedicated to consumers but also for the broader implications on health. Slowing down the oxidation of fats (thus reducing the formation of harmful products from secondary oxidation) or proteins (which significantly impact their functional properties) plays a key role in affecting the color, taste, and nutritional value of food. Moreover, the consumption of foods with high potential against reactive oxygen species can be viewed as a dietary approach to support the body’s natural mechanisms in maintaining its’ oxidation–reduction balance. This preventive measure against oxidative stress is fundamental in the strategy to prevent numerous civilization diseases [25,26,27]. Additionally, scientists are exploring another avenue concerning ’peptides, namely assessing their inhibitory effect on various enzymes. Overproduction of these enzymes may contribute to the occurrence of pathological conditions leading to diseases. For instance, peptides acting as ACE-I inhibitors are being considered as a strategy for treating hypertension [28,29]. Similarly, dipeptidyl peptidase IV inhibitors and alpha-glucosidase inhibitors represent distinct treatment strategies for diabetic patients [29,30]. Numerous scientific reports, based on in vitro or in silico analyses, suggest that food-derived peptides exhibit significant potential, particularly in terms of antioxidant activity and inhibiting the action of these enzymes [31,32,33]. This potential is further supported by the biological activity profile of canned meat peptides obtained in this study, encompassing 77 sequences common to all analyzed variants. Among the 22 different potential effects identified, the most frequently observed include the inhibition of DPP IV (100% of the sequence; exhibiting an antidiabetic effect) and ACE-I (99% of the sequence; demonstrating an antihypertensive effect), along with antioxidant activities (66% sequence; exerting multi-directional action against aging caused by oxidative factors) and alpha-glucosidase inhibition (66% of the sequence; displaying an antidiabetic effect), among others (Figure 4).

The utilization of bioinformatics methods in food research has gained popularity recently, primarily because of the potential to reduce analysis costs and time while enabling a more precise search for peptide sequences with the most beneficial effects. Typically, in vitro studies involve a collective analysis of peptides present in isolates or extracts without isolating a specific factor (peptide) responsible for a particular effect to the greatest extent. However, the integration of in silico methods (sequence identification), followed by the chemical synthesis of a specific peptide, and subsequent assessment of its bioactivity in the laboratory (in vitro) or on living organisms (in vivo), appears to offer a comprehensive approach for discovering new biologically active peptides.

In this study, spectrometric methods were employed to determine the peptide sequences isolated from canned pork meat. The research focused on two selected variants for further investigation: W_C and W_15. Among the numerous peptides identified, 83 sequences were exclusively present in selected samples after 180 days of refrigeration storage, and these were chosen for further consideration. As indicated by the data in Figure 4, the peptides most frequently identified in canned meat fortified with currant extract were those exhibiting antioxidant or enzyme-inhibiting activity. Of particular interest were peptides with inhibitory activity against metabolically important enzymes, although demonstrating their activity in vivo posed significant challenges. Consequently, our further analysis concentrated on three specific activities: peptides capable of inhibiting the action of DPP-IV, ACE-I, and alpha-glucosidase. These peptides underwent thorough in vivo analysis utilizing molecular docking models. It is crucial to note that the mechanisms employed to inhibit the action of these enzymes in pharmacology are integral in treating noncommunicable diseases. Conversely, discovering bioactive compounds of natural origin, such as peptides present in food, holds the potential to mitigate many side effects associated with synthetic drugs. In this context, food could serve as a preventive or therapeutic factor for individuals without negative side effects. The most promising results from the in silico analysis, comprising 10 sequences, are presented in Table 3.

Certainly, within the chosen sequences, the peptides exhibited the highest potential for biological activity, particularly in inhibiting the action of the DPP IV and ACE-I enzymes, with A values surpassing 1. Subsequently, these peptides demonstrated inhibitory effects on alpha-glucosidase and displayed antioxidant properties. The most potent peptide in each category, based on inhibitory activity, was singled out for further investigation to assess its potential action via molecular docking analysis. The minimized 3D structural characteristics of these peptides are detailed in Table 4.

### 2.4. Molecular Docking Analysis

To assess the binding affinity of specific peptides with the proteins 5NN8, 1O86, and 2QT9 within their designated binding pockets, molecular docking was employed [34]. The primary objective of molecular docking is to pinpoint the ligand–receptor complex with the highest binding affinity, aiding in predicting the effectiveness of a drug (peptide) or elucidating the mechanism of action of an enzyme. To achieve this, in addition to the binding sites documented in the literature (Table 1), three different computational software programs were utilized for each analyzed protein to predict potential binding positions on their surface. The initial software, fpocket [35], operated with default settings and identified 41 potential binding sites for 5NN8, 32 binding sites for 1O86, and 115 potential binding sites for 2QT9. The second program, CAVITY software [36], identified 35 potential binding sites for the 5NN8 protein, 26 for the 1O86 protein, and 55 for the 2QT9 protein. Additionally, we utilized the open-source GHECOM software (version 1.0) [37], considering all cavities generated using GHECOM. Combining literature data with calculation results, we identified the 10 most significant binding sites for all the studied receptors. The selection of the cavity was arbitrary and guided by its size, shape, and the size of the peptide molecule, maintaining the original numbering of the pockets for consistency with the initially generated files.

The molecular surface of the receptor, where the peptide was affixed, signifies the optimal binding site determined by the best free energy values (∆Gbinding). Following the selection of binding pockets (10 for each), molecular docking was employed to ascertain the binding affinity of specific peptides with enzymes. The choice of the most favorable option was based on the ∆Gbinding of individual peptides with the binding pockets of the studied protein, and the presentation of the identified highest affinity systems (receptor with docked ligand) is detailed in Table 5.

**Figure 5:**
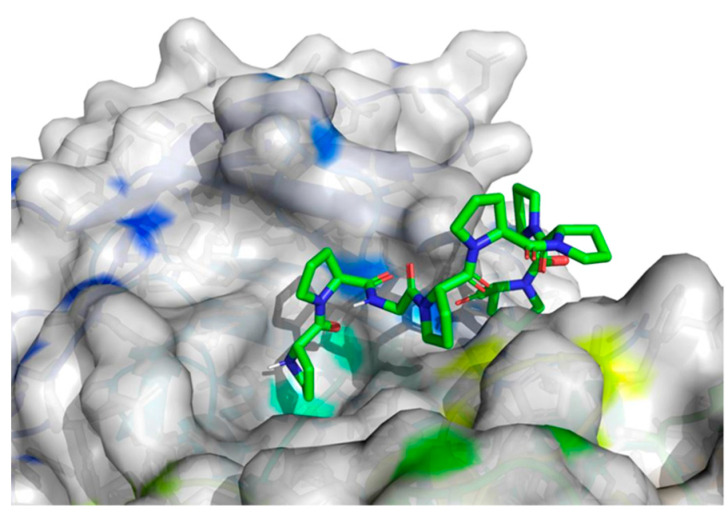
Projection of the best binding pocket of protein PDB ID: 5NN8 with docked peptide of sequence PPGPPPPP.

**Figure 6:**
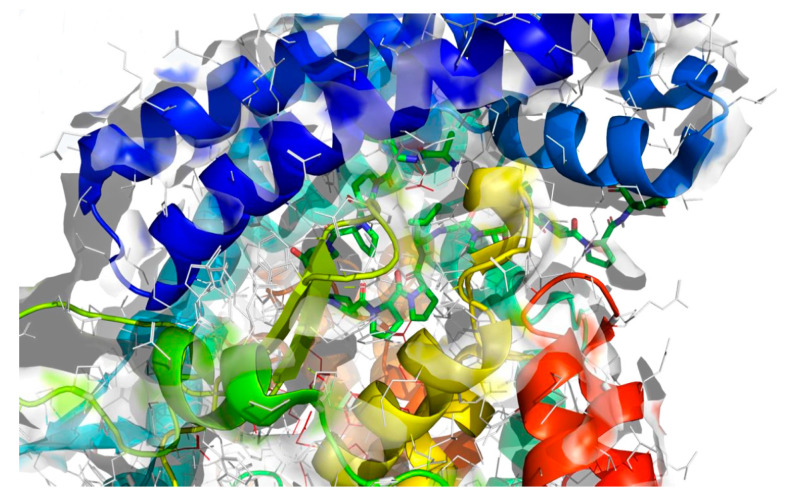
Projection of the best binding pocket of protein PDB ID: 1O86 with docked peptide of sequence.

**Figure 7:**
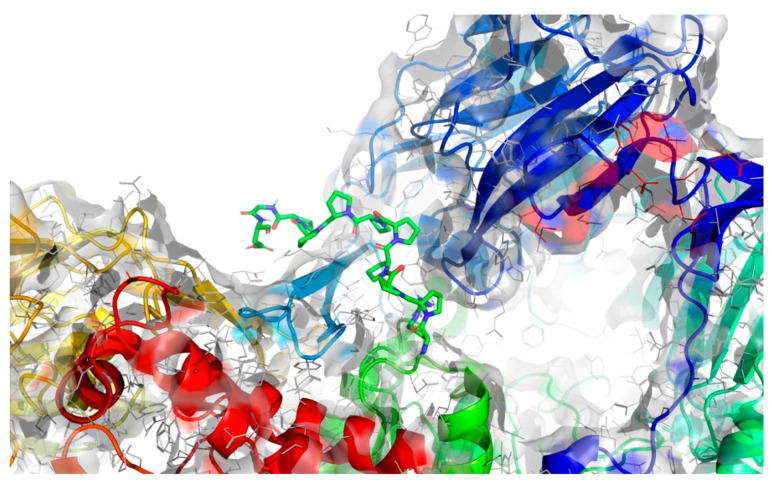
Projection of the best binding pocket of protein PDB ID: 2QT9 with docked peptide of sequence RPPPPPPPPAD.

The best binding sites that were identified on the studied protein surfaces do not fully coincide with the literature data [38,39,40]. However, in the studies described in the cited sources, proteins such as 5NN8, 1O86, and 2QT9 interacted with a completely different ligand in comparison to the studied peptides. Depending on the type of chemical compound, different binding pockets on the protein structure may be preferred. Furthermore, it should be noted that each computational program dedicated to molecular docking is based on different mathematical algorithms and scoring functions, which may lead to certain discrepancies in the resulting data. Figure 5, Figure 6 and Figure 7 depict the spatial conformations of peptides that interact most strongly with the best binding pocket of the studied proteins.

## 3. Materials and Methods

### 3.1. Extract Preparation

The plant material comprised leaves of black currant (*R. nigrum* L.), harvested in May before the bushes entered the flowering stage (Lublin, Poland). The extraction procedure details are outlined in Ferysiuk et al. [20]. In summary, the leaves underwent hot air drying at 60 °C and were then subjected to water extraction (leaf-to-solvent ratio of 1:10 *w*/*v*). Ultrasound assistance was employed during extraction with the following parameters: ultrasound frequency set at 40 kHz, sound intensity at 320 W/cm^2^, and a temperature of 30 °C for 10 min (Sonic 6D equipment, Polsonic Palczynki Sp. J., Warsaw, Poland). The resulting infusions were purified via filtration and subsequently lyophilized for 72 h using a freeze dryer (Free Zone 12 lyophilizer, Labconco Corporation, Kansas City, MO, USA) at −80 °C and 0.04 mbar.

### 3.2. Canned Meat Product Preparation

The product was crafted using a combination of pork shoulder and pork dewlap in a 2:8 ratio. Meats were obtained from an organic farm (Zakład Mięsny Wasąg SP. J., Poland, organic certificate no: PL-EKO-093027/18). Test variants were prepared with a reduced addition of sodium nitrate (50 mg/kg of meat) compared to the control sample (100 mg/kg of meat, W_C). To achieve this, the meat raw material underwent initial grinding with a knife, followed by finer grinding using a meat grinder (universal machine KU2-3E, Mesko-AGD, Skarzysko-Kamienna, Poland). The prepared meat raw material was then divided into individual variants. In this stage, each variant received additions of sodium nitrate, saltwater, and currant leaf extract (refer to Table 6). The addition of water and salt remained consistent across all samples, comprising 5% water and 2% salt, respectively. Subsequently, the meat mixture prepared in this manner underwent a mixing process (5 min/variant; universal machine KU2-3E, Mesko-AGD, Skarzysko-Kamienna, Poland) before being packed into metal cans (300 mL volume). Each can contained approximately 250 g of the mixture. The metal cans were sealed and subjected to the sterilization process in a vertical steam sterilizer (vertical steam sterilizer, TYP-AS2, Warszawa, Poland). Following sterilization, the product was cooled with cold water and stored in a refrigerator (4 °C) for further analysis.

### 3.3. Peptides Isolation and Identification

The peptides were isolated following the procedure outlined by Mora et al. [41]. In summary, samples of canned meat products (15 g) were homogenized with 100 mL of 0.01 N HCl for 5 min and then centrifuged (2200 rpm for 20 min at 4 °C). The resulting supernatant was decanted, filtered through glass wool, and subjected to deproteinization by adding ethanol (at a ratio of 1:3), followed by centrifugation under the previously defined conditions. The obtained supernatant was subsequently dried using a vacuum evaporator (Rotavapor R-215, BüchiLabortechnik AG, Flawil, Switzerland). After concentration in the evaporator, the peptides were dissolved in 2 mL of 0.01 M HCl and subjected to further chromatographic analysis. The analysis of peptides was performed using liquid chromatography coupled with electrospray tandem mass spectrometry (LC–MS/MS) at the Mass Spectrometry Laboratory, following the procedure described by Kęska et al. [33]. Generally, Before the analysis, the samples were concentrated and desalted on an RP-C18 precolumn (Waters Corp., Milford, MA, USA). Separation was performed on an RP-C18 nano-Ultra Performance column (Waters, BEH130 C18 column, 75 μm i.d., 250 mm long) of a nanoACQUITY UPLC system (Warsaw, Poland) using a 180 min linear acetonitrile gradient (0–35%) at a flow rate of 250 nL/min. The column outlet was directly connected to a mass spectrometer (Orbitrap Velos, Thermo Fisher Scientific Inc., Waltham, MA, USA) for the analysis. The raw data files were preprocessed using Mascot Distiller software (version 2.4.2.0, Matrix Science Inc., Boston, MA, USA). The obtained peptide masses and their identified fragmentation pattern were compared with the protein sequence database (UniProt KB) using the Mascot search engine (Mascot Daemon v. 2.4.0, Mascot Server v.2.4.1, Matrix Science, London, UK). The peptide sequences from unknown original proteins were excluded. Peptide identification was performed using the Mascot search engine (Matrix Science) with a probability-based algorithm. The expected value threshold was set at 0.05 for the analysis (all peptide identification had a <0.05% chance of being a random match).

### 3.4. Multifunctional Properties of Processed Meat Peptides with Currant Leaf Extract—In Vivo Analysis

#### 3.4.1. Evaluation of Biofunctionality of Peptides

The search was conducted utilizing the BIOPEP-UWM database [42]. In this process, profiles of potential biological activity were assessed. Subsequently, the “Calculations” tab was employed to determine the frequency of occurrence of bioactive fragments in the protein sequence, as indicated by parameter A. This parameter is contingent on the number of fragments with a specific activity in the protein sequence (*a*) and the total number of protein amino acid residues (N), calculated using the following formula:

(1) A=a/N

In the bioinformatic analysis, online tools from PepDraw [43] were employed to estimate the fundamental physicochemical parameters of the three chosen peptide sequences with the most significant antihypertensive and antidiabetic potential. 

#### 3.4.2. Molecular Docking

**Figure 8:**
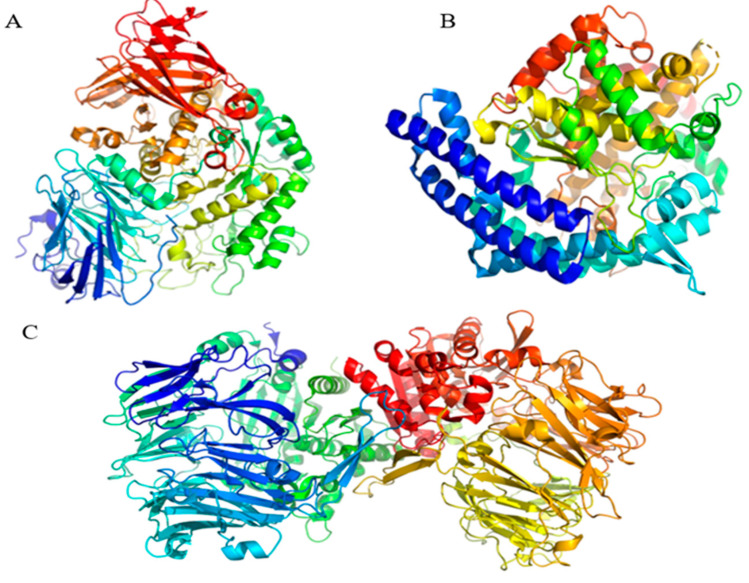
The three-dimensional structure of human lysosomal acid α-glucosidase (PDB ID: 5NN8; (**A**)); angiotensin-converting enzyme (PDB ID: 1O86; (**B**)) and dipeptidyl peptidase IV/CD26 (PDB ID: 2QT9; (**C**)).

The receptor employed in the molecular docking method was chain A of the crystal structure of human lysosomal ac-id-alpha-glucosidase, GAA (PDB ID: 5NN8) [44], chain A of the crystal structure of human angiotensin-converting enzyme, ACE (PDB ID: 1O86) [45], and human dipeptidyl peptidase IV/CD26, DPP4 (PDB ID: 2QT9) (Figure 8).

The initial step involved removing from the structure all entities not constituting a protein receptor that could interfere with the molecular docking process. 

For PDB ID: 5NN8, these entities included water molecules, s-hydroxycysteine, α-L-fucopyranose, 2-acetamido-2-deoxy-β-d-gluxopyranose, β-d-mannopyranose, α-d-glucopyranose, 4,6-dideoxy-4-{[{1*S*, 4*R*, 5*S*, 6*S*)-4,5,6-trihydroxy-3-(hydroxymethyl) cyclohex-2-en-1-yl]amino}-α-d-gluxopyranose, sulfate ions, chloride ions, glycerol, 1,2-ethanediol, triethylene glycol, ethylene glyxol, *N*-[4-hydroxymethyl-cyclohexan-6-yl-1,2,3-triol]-4,6-dideoxy-4-aminoglucopyranoside, and glycerin. 

For PDB ID: 1O86, the ex-cluded entities were water molecules, glycine, Zn^2+^ ion, [N2-[(*S*)-1-carboxy-3-phenylpropyl]-L-lysyl-Lproline, and lisinopril. 

For PDB ID: 2QT9, the excluded entities were water molecules, 2-acetamido-2-deoxy-b-D-glucopyranose, 2-acetamido-2-deoxy-a-d-glucopyranose, Na^+^ ion, (2*S*,3*S*)-3-amino-4-[(3*S*)-3-fluoropyrrolidin-1-yl]-*N*,*N*-dimethyl-4-oxo-2-(trans-4-[1,2,4]triazolo [1,5-A]pyridine-5-yl cyclohex-yl)butanamide, N-acetyl-b-d-glucosaamine, *N*-acetyl-a-d-glucosamine, and 5-(4-{(1*S*,2*S*)-2-ammonio-1-[(dimethylamino)carbonyl]-3-[(3*S*)-3-fluoropyrrolidin-1-yl]-3oxopropyl}cyclohexyl)[1,2,4]triazolo[1,5-A]pyridin-1-ium. The subsequent step involved preparing the receptor for molecular docking, following the previously described procedure [33]. This entailed adding hydrogen atoms and partial charges to the receptor’s structure, as well as optimizing it. To accomplish this, the AutoDockTools package, which is part of the MGLTools software (version 1.5.7) suite, was utilized [34,46]. Hydrogen atoms and partial charges (Gasteiger) were added using this package. Additionally, energy minimization was performed using the General Amber Force Field (GAFF) in the Open Babel software (version 3.0.0) [47]. The file format was also appropriately converted to the one required by the QuickVina-W docking engine [48].

The structures of ligands and their preparation were conducted as outlined in a previous description [33]. Generally, the three-dimensional structures of the peptides were predicted based on their amino acid sequences using the ECEPP software (ECEPP-05 version) [49], using an Electrostatically Driven Monte Carlo (EDMC) method for peptide structure determination. The simulation proceeds via a series of Monte Carlo steps, driven by the electrostatic interaction energy between the charged residues, to pick up different variants of the peptide conformation. Finally, the resulting conformations were ranked based on their energies, and the lowest energy conformations were selected as potential 3D structures for peptides. The generated structures of each peptide were subjected to a short optimization process using the GAFF force field [47]. The subsequent step involved adding partial charges to the ligand structures. Similar to the receptor, the AutoDockTools package was employed for this task. The file format was also converted to the format required by the QuickVina-W docking engine. The quest for the optimal ligand (peptide) binding site on the enzyme molecule’s receptor relied on literature data, as depicted in Table 7. In addition to the binding sites identified in the literature, three distinct computational software types were employed for each analyzed protein to forecast potential binding poses on their surface [33]. We used three different variants of the peptide conformation. Finally, the resulting conformations were ranked based on their energies, and the lowest energy conformations were selected as potential 3D structures for peptides. The generated structures of each peptide were subjected to a short optimization process using the GAFF force field [47]. The subsequent step involved adding partial charges to the ligand structures. Similar to the receptor, the AutoDockTools package was employed for this task. The file format was also co-docking analyses were executed for the ten selected cavities, with each of the four designated ligands being individually docked into every cavity (Supplementary Materials).

### 3.5. Antioxidant Properties of Peptides from Meat Products with Currant Leaf Extract—In Vitro Analysis

#### 3.5.1. The Ability to Neutralize the ABTS Radicals

The capacity of the extracted meat samples to counteract free radicals was assessed following the method described by Re et al. [50], employing the ABTS^•^* free radical. The extent of ABTS^•^* reduction was measured spectrophotometrically at a wavelength of 734 nm. The antiradical capability, indicating the ability to neutralize free radicals, was determined using the following formula:

(2) $$ABTS\;[\%]=[1-(A_{2}/A_{1})]\times 100$$

where *A*_1_—absorbance of the control sample; *A*_2_—absorbance of the specific sample.

#### 3.5.2. Ability to Chelate Iron (II) Ions

The chelating capacity of iron (II) ions by bioactive compounds present in extracts from canned meat products was assessed according to the method of Decker and Welch [51]. The absorbance of the colored complex was measured spectrophotometrically at 562 nm according to the following formula:

(3) $$Chelate\;iron\;(II)\;ions\;[\%]=[1-(A_{2}/A_{1})]\times 100$$

where *A*_1_—absorbance of the control sample; *A*_2_—absorbance of the specific sample.

#### 3.5.3. Ability to Reduce Iron (III) Ions (Reducing Power)

The assessment of the reduction power, following the method of Oyaizu [52], involves the reduction of the reactant (Fe^3+^) in stoichiometric excess relative to the antioxidants. This spectrophotometric method is performed at 700 nm, where a higher absorbance value indicates a greater reducing capacity of the test sample.

### 3.6. Statistical Analysis

Statistical analysis was conducted using Statistica software (https://statistica.software.informer.com/) and Microsoft Office Excel 2013. All laboratory analyses were carried out in triplicate, and the results are expressed as means (±standard deviation). Homogeneous groups were distinguished using Tukey’s test, with differences deemed statistically significant at *p* < 0.05. Graphical representation of the relationships between the samples based on antioxidant tests was accomplished via hierarchical data grouping. The results were visualized using a dendrogram, employing the Ward method as the agglomeration technique and the Euclidean distance as the distance measure. Owing to the specific functional characteristics of canned meat, designed for extended storage with a prolonged shelf life, the results of in vivo and molecular docking analyses were presented only after 180 days of refrigerated storage.

## 4. Conclusions

The results presented in this study confirm the potential of canned meat as a functional food attributed to the biological effects of the ingredients they contain, especially peptides. It has been shown that the addition of freeze-dried black currant extract to canned meat in the amount of 150 mg/kg increases the antioxidant value of canned meat. In silico studies have also shown that such technological treatments promote the production of peptides with other key activities in the treatment of people with dietary ailments, such as hypertension or diabetes. This knowledge should be compared in vitro and in vivo to confirm the functional nature of canned pork with the addition of blackcurrant leaf extract and with a low nitrate content.

## Figures and Tables

**Table 1:** Comparison of peptide content based on spectrometric analyses.

Storage Time [Days]	Number of Identified Peptide Sequences			
	W_C	W_05	W_10	W_15
1	530	1129	584	714
180	385	1188	821	900

W_C—control; W_05—black currant leaves extract 50 mg/kg; W_10—black currant leaves extract 100 mg/kg; W_15—black currant leaves extract 150 mg/kg.

**Table 2:** Test results of the antioxidant properties of the analyzed samples (*n* = 9).

TEST	Time [Day]	W_C	W_05	W_10	W_15
ABTS [%]	1	23.13 ± 2.26 ^Bd^	46.07 ± 1.84 ^Bb^	36.43 ± 2.59 ^Bc^	50.97 ± 1.48 ^Ba^
	180	27.42 ± 1.02 ^Ac^	56.81 ± 2.61 ^Ab^	57.07 ± 3.70 ^Ab^	67.36 ± 2.72 ^Aa^
Chelate iron (II) ions [%]	1	15.68 ± 1.39 ^Bb^	16.98 ± 1.81 ^Bb^	20.24 ± 1.48 ^Ba^	21.86 ± 0.95 ^Ba^
	180	21.41 ± 1.65 ^Ac^	24.19 ± 0.29 ^Ac^	28.89 ± 1.40 ^Ab^	46.09 ± 3.23 ^Aa^
Reduction power [A700]	1	1.79 ± 0.006 ^Ba^	1.74 ± 0.08 ^Ba^	1.64 ± 0.03 ^Bb^	1.55 ± 0.02 ^Bc^
	180	1.80 ± 0.008 ^Ac^	2.05 ± 0.05 ^Aa^	1.96 ± 0.05 ^Ab^	1.81 ± 0.05 ^Ac^

W_C—control; W_05—black currant leaves extract 50 mg/kg; W_10—black currant leaves extract 100 mg/kg; W_15—black currant leaves extract 150 mg/kg. Means with different capital letters are significantly different (*p* < 0.05) in the same column. Means with different small letters are significantly different (*p* < 0.05) in the same row. Results are presented as mean ± SD.

**Table 3:** List of peptides with the highest selected biological activity.

No.	Peptides Sequence	*A* Parameter
	DPP IV inhibiting activity	
1	**RPPPPPPPPAD** ^1^	1.364
2	PPPGPPPPGPPPPGPAPPGARPPPGPPPPGPPPPGP	1.278
3	PPPGPAPPGARPPPGPPPPGPPPPGPAPPGARPPPGPPPPGPPPPGP	1.255
4	PPGPPPPP	1.250
5	PPGPAPPGARPPPGPPPPGPPPPGPAPPGARPPPGPPPP	1.231
6	PPPGPAPPGARPPPGPPPPGPPPPGP	1.231
7	APPGARPPPGPPPPPPGPSPPRPPPGPPPQ	1.133
8	YQEPVLGPVRGPFPILV	1.118
9	KPKKKPPPPAGPPPPGPPSPGP	1.091
10	APPGARPPPPPPPPADEPQQGPAPSGDKPKKKPPPPAGPPPPGPPSPGP	1.082
	ACE-I inhibiting activity	
1	**ARPPPGPPPLGPPPPGP**	1.529
2	PPPGPPPPGPPPPGPAPPGARPPPGPPPPGPPPPGP	1.417
3	PPPGPAPPGARPPPGPPPPGPPPPGPAPPGARPPPGPPPPGPPPPGP	1.383
4	PGPPPPP	1.375
5	RPPPPPPPPAD	1.364
6	PPGPAPPGARPPPGPPPPGPPPPGPAPPGARPPPGPPPP	1.359
7	PPPGPAPPGARPPPGPPPPGPPPPGP	1.346
8	APPGARPPPGPPPPPPGPSPPRPPPGPPPQ	1.267
9	YQEPVLGPVRGPFPIIV	1.059
10	LLYQEPVLGPVRGPFPIIV	1.053
	Alpha-glucosidase inhibiting activity	
1	**PPGPPPPP**	0.625
2	PPPGPPPPGPPPPGPAPPGARPPPGPPPPGPPPPGP	0.472
3	APPGARPPPGPPPPPPGPSPPRPPPGPPPQ	0.433
4	PPPGPAPPGARPPPGPPPPGPPPPGPAPPGARPPPGPPPPGPPPPGP	0.426
5	PPPGPAPPGARPPPGPPPPGPPPPGP	0.423
6	ARPPPGPPPLGPPPPGP	0.412
7	PPGPAPPGARPPPGPPPPGPPPPGPAPPGARPPPGPPPP	0.410
8	APPGARPPPPPPPPADEPQQGPAPSGDKPKKKPPPPAGPPPPGPPSPGP	0.326
9	KPKKKPPPPAGPPPPGPPSPGP	0.318
10	RPPPGGGPPRPPPPEESQGEGHQKRPRPPGDGPEQGP	0.243
	Antioxidative activity	
1	TLWGIQKDLKDL	0.500
2	LLYQEPVLGPVRGPFPIIV	0.263
3	LLYQEPVLGPVRGPFPILV	0.211
4	KPKKKPPPPAGPPPPGPPSPGP	0.182
5	YQEPVLGPVRGPFPIIV	0.177
6	PLNETVVGLYQK	0.167
7	AAWQKLTNAVANALAHKYH	0.158
8	AIRGDEELDSLIKATIAGGGVIPHIH	0.154
9	PLNETVVGLYQKS	0.154
10	LNLPTGIPIVYEL	0.154

^1^ sequences selected for further analysis are bolded.

**Table 4:** Minimized 3D structure of a peptide with the sequence RPPPPPPPPAD, ARPPPGPPPLGPPPPGP, and PPGPPPPP and their characteristic.

Peptide Sequences	Peptides 3D Structure	Peptides Characteristic ^1^		Receptor (PDB ID)
RPPPPPPPPAD	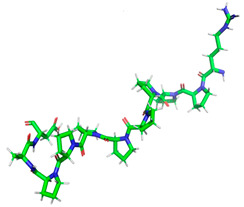	Length: Mass: Isoelectric point: Net charge: Hydrophobicity:	11 1136.596 6.80 0 +14.97 Kcal * mol ^−1^	2QT9
ARPPPGPPPLGPPPPGP	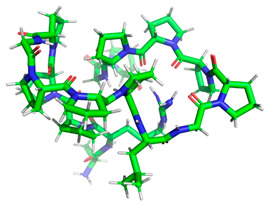	Length: Mass: Isoelectric point: Net charge: Hydrophobicity:	17 1596.875 11.56 +1 +19.95 Kcal * mol ^−1^	1O86
PPGPPPPP	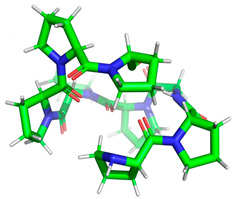	Length: Mass: Isoelectric point: Net charge: Hydrophobicity:	8 754.400 5.25 0 +10.03 Kcal * mol ^−1^	5NN8

^1^ results obtained using PepDraw tools.

**Table 5:** The best identified binding regions on the molecular surface of 5NN8, 1O86, and 2QT9 receptors. For each binding site, the amino acid residues that comprise it, as well as the best free energy values (∆Gbinding) for peptide binding with molecules, have been specified.

Ligand–Receptor 3D Structure	∆Gbinding [kcal/mol]
The best identified binding regions on the molecular surface of 5NN8 receptor with PPGPPPPP ^1^	
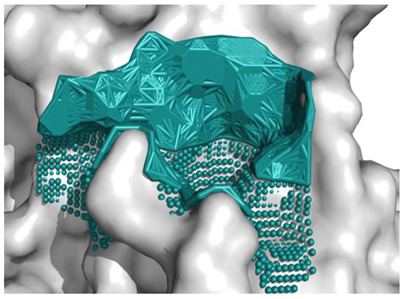	−8.4
The best identified binding regions on the molecular surface of 1O86 receptor with ARPPPGPPPLGPPPPGP ^2^	
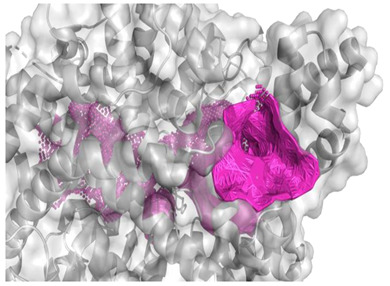	−9.6
The best identified binding regions on the molecular surface of 2QT9 receptor with RPPPPPPPPAD ^3^	
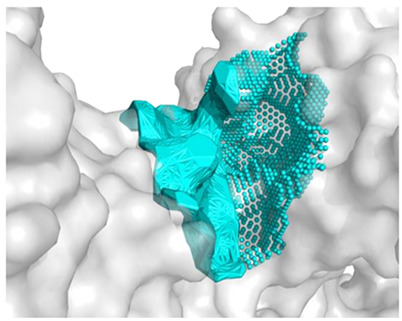	−9.1

^1^ The binding pocket is formed by the following amino acid residues: Pro161, Lys162, Asp163, Ile164, Leu165, Thr166, Lys184, Asp185, Ala187, Asn188, Arg189, Arg190, Tyr191, Glu192, Val193, Pro194, Leu195, Phe241, Ala242, Asp243, Gln244, Asn316, Thr333, Gly334, Gly335, Ile336, Thr491, Asn536, Glu537, Leu538, Glu539, Ala559, Ser560, Ser561, His562, Gln563, Phe564, Leu565, Ser566, Thr567, His568, Tyr569, Asn570, Leu571, Leu574. ^2^ The binding pocket is formed by the following amino acid residues: Arg100, Lys101, Val104, Lys113, Arg114, Ile115, Ile116, Lys117, Lys118, Val119, Gln120, Asp121, Leu122, Glu123, Arg124, Ala125, Ala126, Leu127, Leu132, Tyr135, Asn136, Lys137, Ile138, Leu139, Leu140, Asp141, Met142, Glu143, Thr144, Thr145, Tyr146, Ser147, Val148, Ala149, Thr150, Leu159, Gln160, Leu161, Glu162, Pro163, Asp164, Leu165, Thr166, Asn167, Val168, Met169, Ala170, Thr171, Ser172, Arg173, Trp185, Ala189, Tyr200, Leu203, Ile204, Asn205, Gln206, Ala207, Ala208, Arg209, Leu210, Asn211, Gly212, Tyr213, Val214, Asp215, Ala216, Gly217, Asp218, Ser219, Trp220, Arg221, Ser222, Met223, Tyr224, Glu225, Thr226, Pro227, Leu229, Leu275, Gly276, Asn277, Met278, Trp279, Gln281, Thr282, Trp283, Ser284, Asn285, Ile286, Tyr287, Asp288, Leu289, Pro297, Ser298, Met299, Asp300, Thr301, Thr302, Glu303, Ala304, Met305, Leu306, Lys343, Pro344, Thr345, Asp346, Gly347, Arg348, Glu349, Val350, Val351, Cys352, His353, Ala354, Ser355, Ala356, Trp357, Asp358, Phe359, Tyr360, Arg366, Lys368, Gln369, Cys370, Thr371, Thr372, Val373, Asn374, Leu375, Glu376, Asp377, Leu378, Val379, Val380, Ala381, His382, His383, Glu384, His387, Ile388, Tyr390, Phe391, Tyr394, Val399, Ala400, Leu401, Arg402, Glu403, Gly404, Ala405, Asn406, Pro407, Gly408, Phe409, His410, Glu411, Gly414, Asp415, Ala418, Leu419, Val421, Ser422, Thr423, Pro424, Leu427, Leu433, Asn445, Phe446, Met448, Lys449, Met450, Ala451, Leu452, Asp453, Lys454, Phe457, Phe460, Phe472, Tyr51, Ala510, Lys511, Phe512, His513, Ile514, Pro515, Ser516, Ser517, Val518, Pro519, Tyr520, Arg522, Tyr523, Ser526, Phe527, Gln530, Ser55, Phe570, Pro573, Val58, Trp59, Asn60, Glu61, Tyr62, Ala63, Glu64, Asn66, Trp67, Tyr69, Asn70, Ile73, Ser78, Leu81, Leu82, Lys84, Asn85, Met86, Gln87, Ile88, Ala89, Asn90, His91, Thr92, Leu93, Tyr95, Gly96, Ala99. ^3^ The binding pocket is formed by the following amino acid residues: Asn119, Tyr120, Val121, Lys122, Gln123, Trp124, Arg125, Ser127, Tyr128, Thr129, Ala130, Ser131, Asn150, Asn151, Thr152, Gln153, Val167, Trp168, Asn169, Asn170, Asp171, Gly189, Lys190, Glu191, Asp192, Ile193, Ile194, Tyr195, Ile198, Thr199, Asp200, Trp201, Val202, Glu204, Tyr211, Asp230, Thr231, Val233, Pro234, Leu235, Ile236, Glu237, Tyr238, Ser239, Phe240, Tyr241, Ser242, Asp243, Leu246, Tyr248, Pro249, Lys250, Thr251, Val252, Arg253, Val254, Pro255, Tyr256, Thr706, Ala707, Asp708, Asp709, Asp737, Glu738, Asp739, His740.

**Table 6:** Variants of meat products.

Sample	Extract [mg/kg]	Sodium Nitrate [mg/kg]
W_05	50	50
W_10	100	50
W_15	150	50
W_C (control)	-	100

**Table 7:** List of regions where binding poses for the receptor are located on the molecule.

Molecules	The Binding Pose for the Using Receptor is Situated in the Following Regions	References
α-glucosidase (PDB ID: 5NN8)	Trp376, Tyr378, Leu405, Trp481, Asp518, Met519, Phe525, Asp616, Trp618, Phe649, Leu650, His674, and Leu678.	[38]
Angiotensin Converting Enzyme (PDB ID: 1O86)	Ala354, Glu384, Tyr523, Gln281, His353, Lys511, His513, Tyr520, Glu162, His383, His387, Glu411, Ala356, Arg522, Glu123, Asp377, Glu376, Asp377	[39]
Dipeptidyl Peptidase IV/CD26 (PDB ID: 2QT9)	Ser101, Ile102, Glu91, Asn92, Ser93, Thr94, Phe95, Asp96	[40]

## Data Availability

Data are contained within the article and supplementary materials.

## Supplementary Materials

The following supporting information can be downloaded at https://www.mdpi.com/article/10.3390/molecules28248009/s1.
