# Supplementary material for: Biological Activity of Canned Pork Meat Fortified Black Currant Leaf Extract: In Vitro, In Silico, and Molecular Docking Study
Source: Molecules. 2023 Dec 8;28(24):8009. doi: 10.3390/molecules28248009 (PMC10745298; doi:10.3390/molecules28248009)
Supplement: Supplementary file 1 [file molecules-28-08009-s001.zip › molecules-2704813-supplementary.pdf]

Table S1. The best 10 identified binding regions on the molecular surface of 5NN8 receptor. **For each binding site, the amino acid residues that comprise it have been specified**

No	Results	Free energy values ΔGbinding [kcal/mol]
1	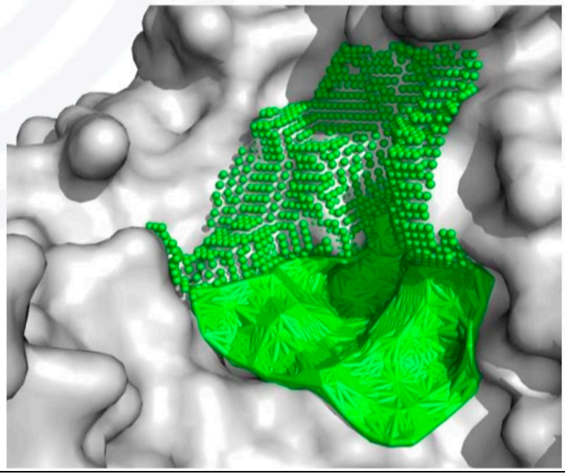<br>The binding pocket is formed by the following amino acid residues: Glu346, Pro347, Lys348, Ser349, Val350, Gln352, Tyr360, His708, Thr711, Leu712, Phe713, His714, Gln715, Ala716, Val718, Ala719, Gly720, Glu721, Thr722, Val723, Arg725, Leu729, Glu730, Phe731, Pro732, Lys733, Trp746, Gly747, Glu748, Ala749, Leu750, Leu769, Gly770, Thr771, Leu818, Arg819, Ala820, Gly821, Tyr822, Ile823, Ile824, Pro825, Ala846, Leu847, Thr848, Gly851, Glu852, Ala853,Arg854, Gly855, Glu856, Leu857, Phe858, Leu868, Tyr873, Gln875, Val876, Ile877, Phe878, Leu879, Ala880, Arg881, Val890	-6.5
2	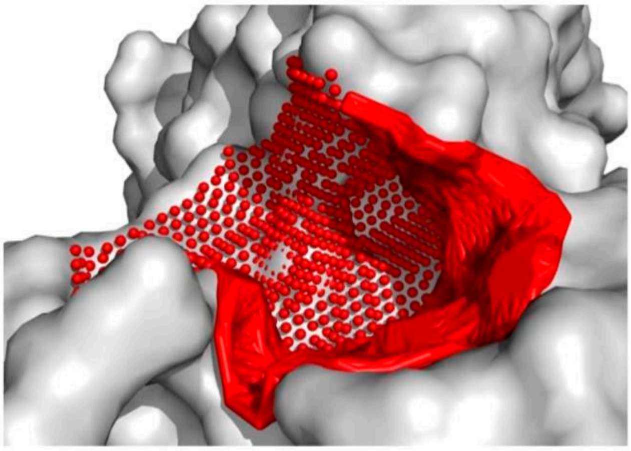<br>The binding pocket is formed by the following amino acid residues: Glu196, Gln352, Tyr354,Leu355, Asp356, Val357, Val358, Gly359, Tyr360, Pro361, Phe362, Met363, Pro364, Pro365,Ile581, His584, Arg585, Ala586, Leu587, Val588, Lys589, Gly592, Thr593, Arg594, Pro595, Gly607, Arg608, Tyr609, Tyr710, Phe713, His714, His717, Val718, Ala719, Gly720, Phe858, Asp860, Glu863, Ser864, Leu865, Glu866, Val867, Leu868, Glu869, Arg870, Ala872, Tyr873	-7.5

3	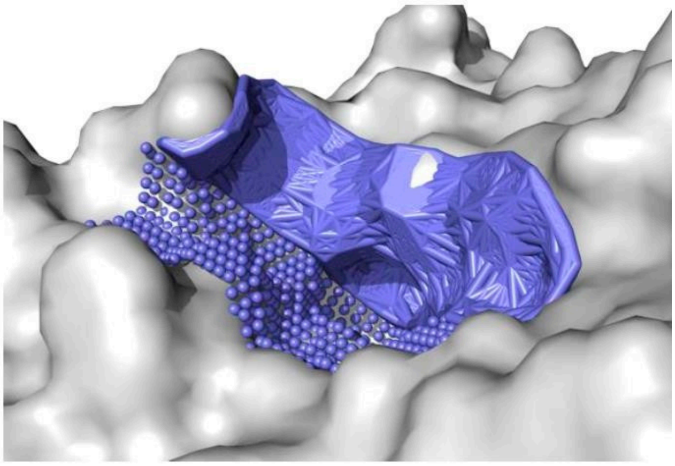	-6.8
	The binding pocket is formed by the following amino acid residues: Arg281, Asp282, Leu283,Ala284, Pro285, Tyr292, Trp376, Asp404, Leu405, Arg411, Ile441, Asp443, Lys479, Trp481, Trp516, Asp518, Met519, Ser523, Asn524, Phe525, Ile526, Ala554, Ala555, Thr556, Arg600,<br>Trp613, Gly615, Asp616, Val617, Trp618, Asp645, Phe649, Leu650, Gly651, Asn652, Arg672,His674, Asn675, Ser676, Leu677, Leu678, Ser679	
4	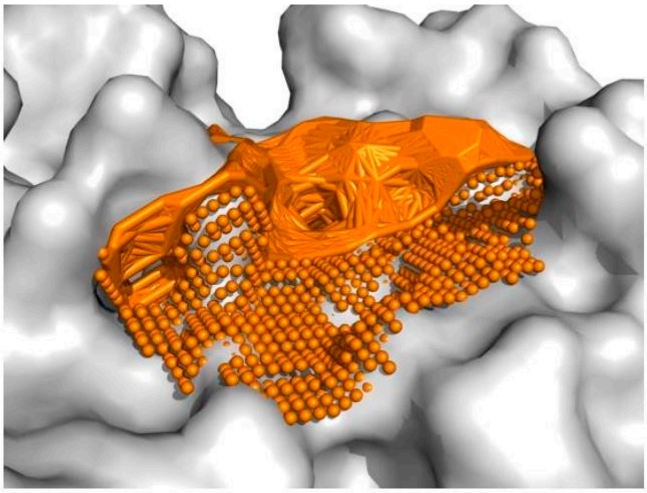	-6.3
	The binding pocket is formed by the following amino acid residues: Met146, Tyr148, Arg168,Leu169, Asp170, Val171, Met172, Met173, Glu174, Thr175, Arg178, His180, Phe181, Thr182,Ile183, Lys184, Arg189, Arg190, Tyr191, Glu192, Val193, Pro194, Leu195, Glu196, Leu246, Leu312, Leu313, Asn314, Ser315, Asn316, Ser332, Gly334, Gly335, Ile336, Leu337, Asp338, Tyr340, Gln353, Leu355, Asp356, Val357, Val358, Gly359, Tyr360, Asn570, Gly605, Arg608,<br>Tyr609	
5	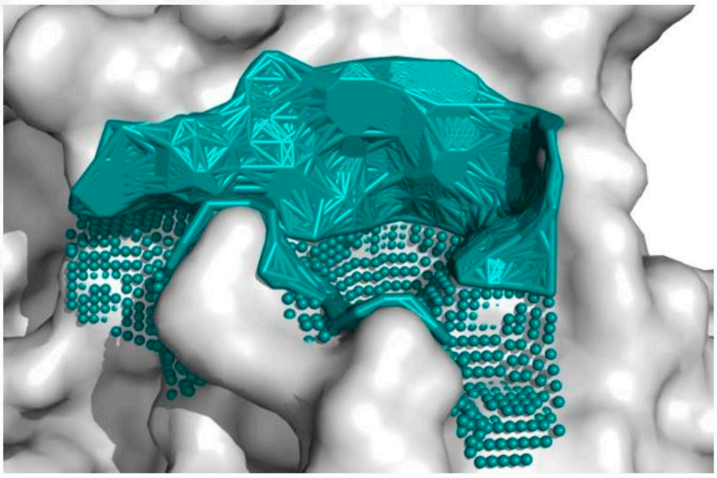	-8.4

	The binding pocket is formed by the following amino acid residues: Pro161, Lys162, Asp163,Ile164, Leu165, Thr166, Lys184, Asp185, Ala187, Asn188, Arg189, Arg190, Tyr191, Glu192, Val193, Pro194,Leu195, Phe241, Ala242, Asp243, Gln244, Asn316, Thr333, Gly334,Gly335, Ile336, Thr491, Asn536, Glu537, Leu538, Glu539, Ala559, Ser560, Ser561, His562, Gln563,	

	Phe564, Leu565, Ser566, Thr567, His568, Tyr569, Asn570, Leu571, Leu574	
6	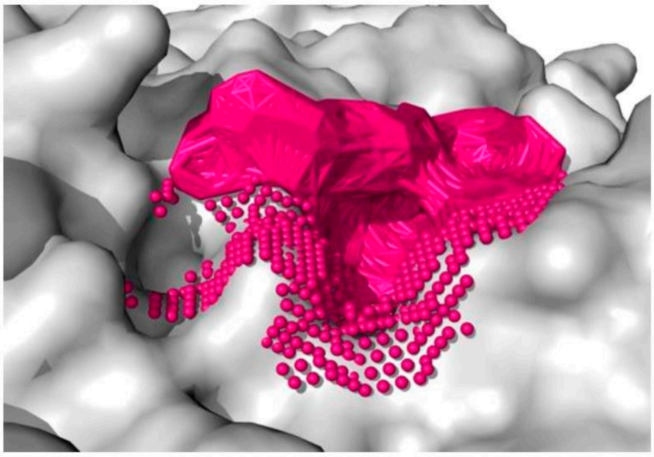	-7.5
	The binding pocket is formed by the following amino acid residues: Gly123, Gln255, Ile257,Thr258, Gly259, Leu260, Ala261, Glu262, His263, Leu264, Ser265, Pro266, Leu267, Met268,Leu269, Ser270, Thr271, Ser272, Trp273, Thr274, Arg275, Ile276, Thr277, Leu278, Thr286, Pro287, Gly288, Ala289, Asn290, Leu291, Asp319, Val320, Leu322, Pro545, Gly546, Val547, Val548, Glu622, Gln623, Ala625, Ser626, Val628, Pro629, Glu630, Ile631, Leu632, Gln633, Phe634, Leu637, Thr739, Asp741, His742	
7	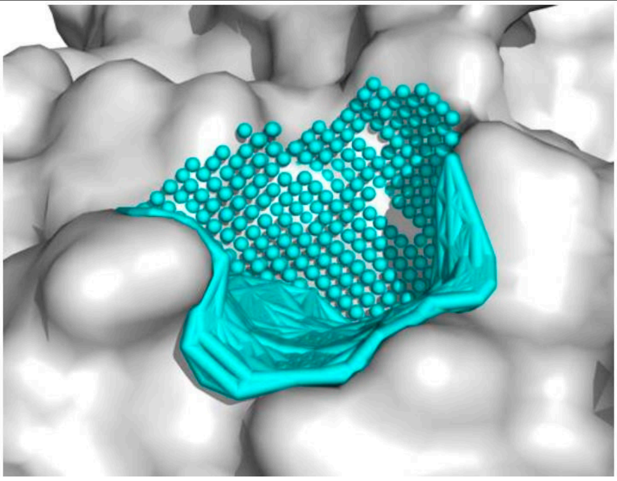	-5.3
	The binding pocket is formed by the following amino acid residues: Trp376, Gly377, Tyr378,Ser379, Ser380, Asp404, Leu405, Asp406, Tyr407, Met408, Asp409, Ser410, Arg411, Arg412,Phe416, Asn417, Lys418, Asp419, Gly420, Phe421, Trp481, Leu677	
8	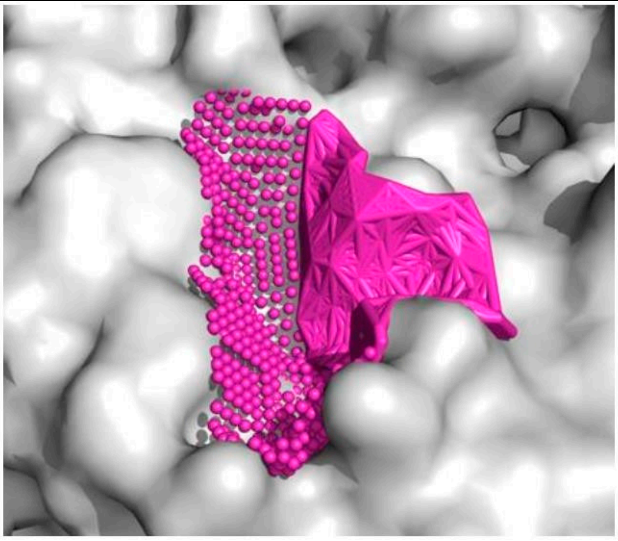	-6.8
	The binding pocket is formed by the following amino acid residues: Pro266, Trp621, Glu622,Ala625, Ser626, Ser736, Thr737, Trp738, Thr739, Val740, Asp741, His742, Gln743, Ile752, Thr753, Pro754, Val755, Leu756, Gln757, Ala758, Lys760, Ala761, Glu762, Val763,	

	Thr764,<br>Gly765, Tyr766, Trp804, Thr806, Leu807, Ala809	

9	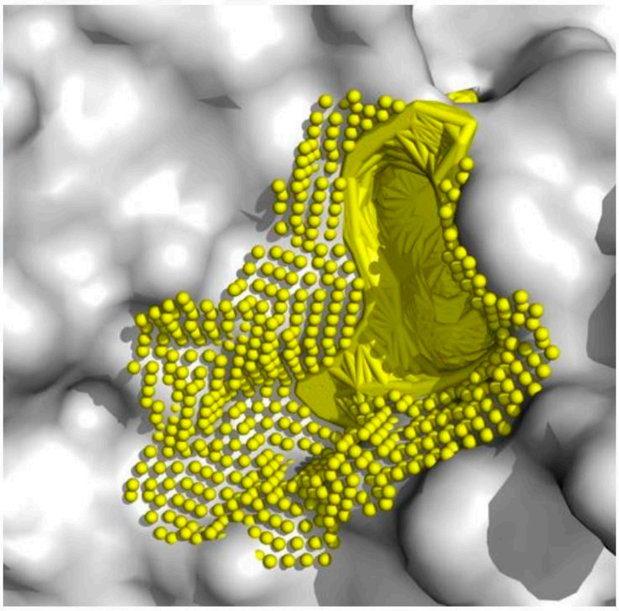<br>The binding pocket is formed by the following amino acid residues: Phe128, Phe129, Pro130, Pro131, Ser132, Tyr133, Pro134, Ser135, Arg154, Ser214, Glu216, Pro217, Phe218, Val230, Asn233, Thr234, Thr235, Val236, Ala237, Pro238, Leu239, Thr250, Ser251, Leu252, Pro253, Ser254, Gln255, Gln323, Pro324, Ser325, Pro326, Ala327, Leu328, Gln81, Cys82, Asp83, Val84, Pro85, Asn87, Ser88, Arg89, Phe90	-6.3
10	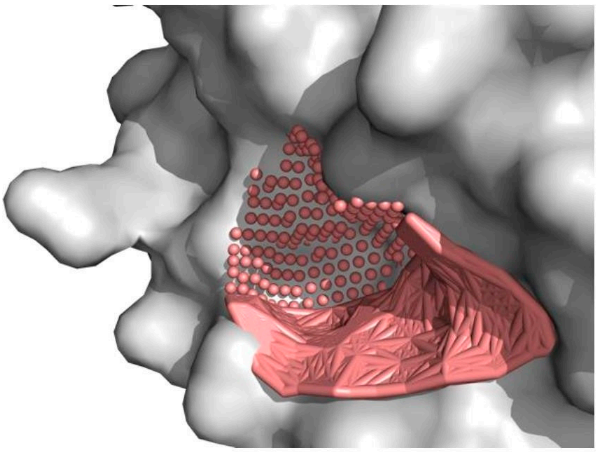<br>The binding pocket is formed by the following aminoacid residues: Arg375, Trp376, Gly377,Tyr378, Ser379, Ala382, Ile383, Thr384, Arg385, Gln386, Val387, Val388, Asn390, Asp406, Asn675, Ser676, Leu677, Leu678, Ser679, Leu680, Pro681, Gln682, Glu683, Tyr685, Ser686, Phe687	-2.1

Table S2. The best 10 identified binding regions on the molecular surface of 1O86 receptor. **For each binding site, the amino acid residues that comprise it have been specified**

No	Results	Free energy values ΔGbinding [kcal/mol]
1	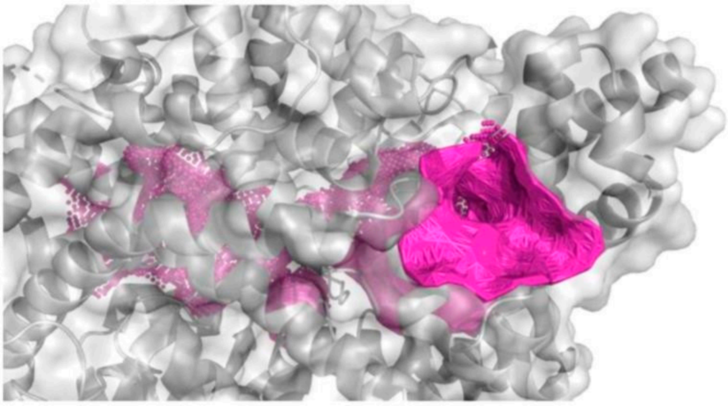 The binding pocket is formed by the following amino acid residues: Arg100, Lys101, Val104, Lys113, Arg114, Ile115, Ile116, Lys117, Lys118, Val119, Gln120, Asp121, Leu122, Glu123, Arg124,Ala125, Ala126, Leu127, Leu132, Tyr135, Asn136, Lys137, Ile138, Leu139, Leu140, Asp141, Met142, Glu143, Thr144, Thr145, Tyr146, Ser147, Val148, Ala149, Thr150, Leu159, Gln160, Leu161, Glu162, Pro163, Asp164, Leu165, Thr166, Asn167, Val168, Met169, Ala170, Thr171, Ser172, Arg173, Trp185, Ala189, Tyr200, Leu203, Ile204, Asn205, Gln206, Ala207, Ala208, Arg209, Leu210, Asn211, Gly212, Tyr213, Val214, Asp215, Ala216, Gly217, Asp218, Ser219, Trp220, Arg221, Ser222, Met223, Tyr224, Glu225, Thr226, Pro227, Leu229, Leu275, Gly276, Asn277, Met278, Trp279, Gln281, Thr282, Trp283, Ser284, Asn285, Ile286, Tyr287, Asp288, Leu289, Pro297, Ser298, Met299, Asp300, Thr301, Thr302, Glu303, Ala304, Met305, Leu306, Lys343, Pro344, Thr345, Asp346, Gly347, Arg348, Glu349, Val350, Val351, Cys352, His353, Ala354, Ser355, Ala356, Trp357, Asp358, Phe359, Tyr360, Arg366, Lys368, Gln369, Cys370, Thr371, Thr372, Val373, Asn374, Leu375, Glu376, Asp377, Leu378, Val379, Val380, Ala381, His382, His383, Glu384, His387, Ile388, Tyr390, Phe391, Tyr394, Val399, Ala400,Leu401, Arg402, Glu403, Gly404, Ala405, Asn406, Pro407, Gly408, Phe409, His410, Glu411, Gly414, Asp415, Ala418, Leu419, Val421, Ser422, Thr423, Pro424, Leu427, Leu433, Asn445, Phe446, Met448, Lys449, Met450, Ala451, Leu452, Asp453, Lys454, Phe457, Phe460, Phe472, Tyr51, Ala510, Lys511, Phe512, His513, Ile514, Pro515, Ser516, Ser517, Val518, Pro519, Tyr520, Arg522, Tyr523, Ser526, Phe527, Gln530, Ser55, Phe570, Pro573, Val58, Trp59, Asn60, Glu61,Tyr62, Ala63, Glu64, Asn66, Trp67, Tyr69, Asn70, Ile73, Ser78, Leu81, Leu82, Lys84, Asn85, Met86, Gln87, Ile88, Ala89, Asn90, His91, Thr92, Leu93, Tyr95, Gly96, Ala99	-9.6

2	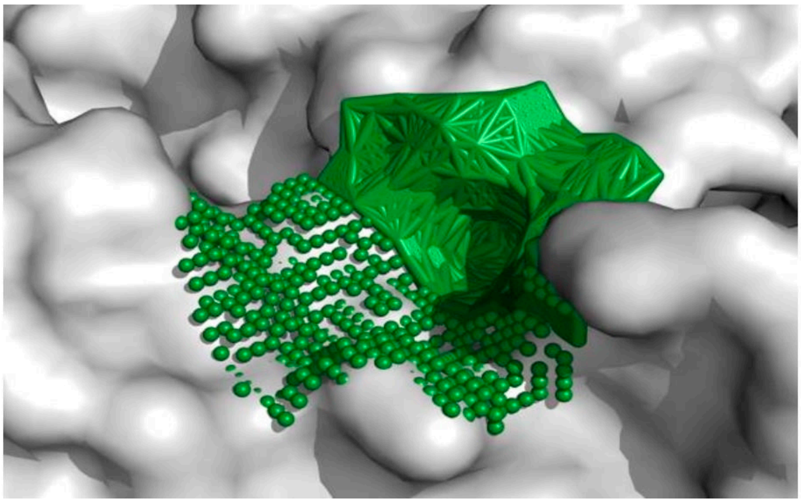	63.1

	The binding pocket is formed by the following amino acid residues: Gln160, Leu161, Glu162,Pro163, Asp164, Asn167, Thr302, Leu306, Trp310, Thr311, Pro312, Arg313, Leu341, Glu342, Lys343, Pro344, Thr345, Cys352, Gln369, Cys370, Thr371, Thr372, Val373, Asn374, Asp377	
3	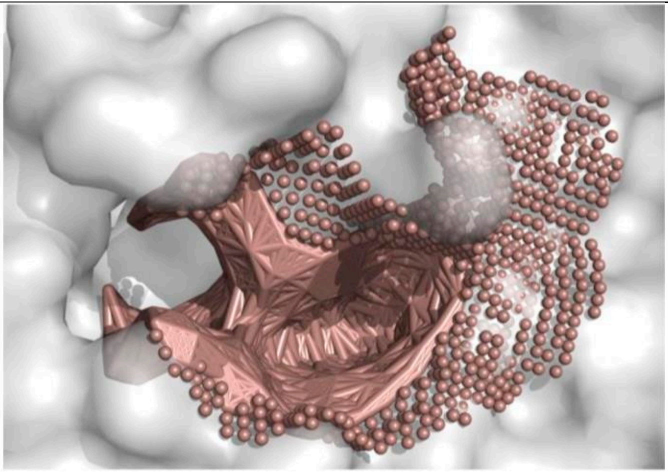	
	The binding pocket is formed by the following amino acid residues: Tyr287, Val291, Phe293, Ala296, Pro297, Ser298, Met299, Thr301, Ser420, Ser422, Thr423, Pro424, Lys425, His426, Leu427, His428, Ser429, Leu430, Leu433, Ser434, Ser439, Asp440, Glu441, His442, Asp443, Ile444, Asn445, Phe446, Leu447, Lys449, Cys538, His543, Thr544, Gly545, Pro546, Leu547, His548, Lys549, Cys550, Asp551, Ile552, Tyr553, Gln554, Tyr595	
4	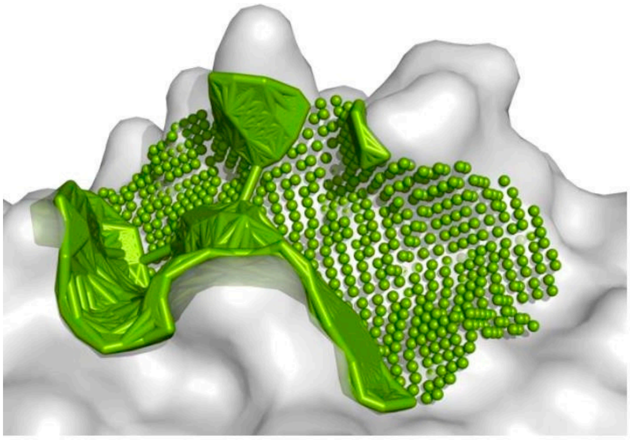	-4.6
	The binding pocket is formed by the following amino acid residues: Ala129, Gln130, Leu132,Glu133, Glu134, Tyr135, Asn136, Lys137, Ile138, Leu140, Asp141, Met142, Glu143, Thr144, Thr145, Val148, Phe196, Glu349, Tyr69, Asn72, Ile73, Thr74, Thr75, Glu76, Thr77, Ser78, Lys79, Ile80, Leu81, Leu82, Gln83, Lys84, Asn85, Met86, Gln87	
5	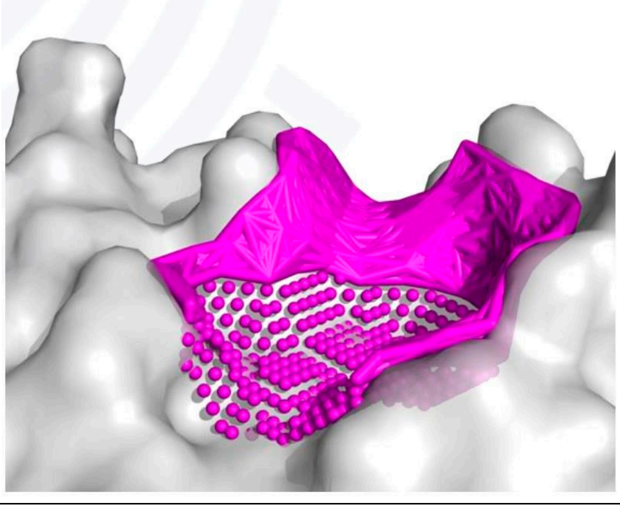	16.1

	The binding pocket is formed by the following amino acid residues: Glu134, Ile138, Leu194,Gln195, Phe196, Tyr197, Pro198, Lys199, Tyr200, Val201, Glu202, Leu203, Ile204, Asn205,<br>Gln206, Arg209, Val214, Asp215, Ala216, Arg470, Val471, Phe472, Asp473, Gly474, Ser475,Ile476, Thr477, Lys478, Glu479, Tyr481	

6	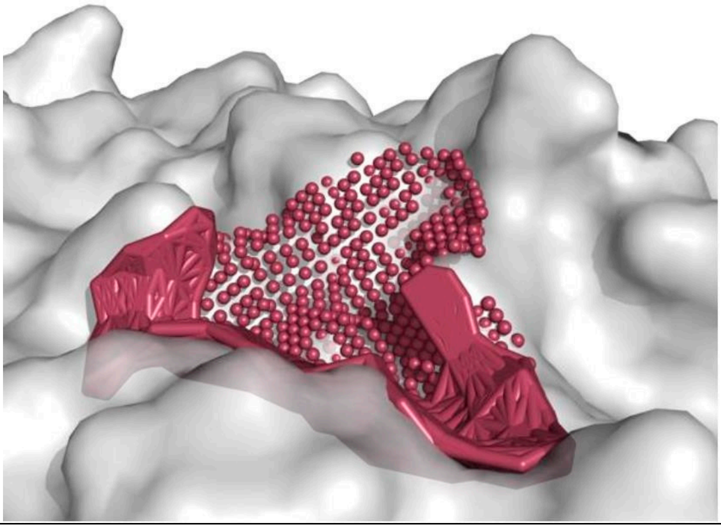<br>The binding pocket is formed by the following amino acid residues: Val214, Asp215, Gly217, Asp218, Arg221, Ser228, Leu229, Glu230, Gln231, Asp232, Leu233, Glu234, Arg235, Gln238, Tyr462, Asp465, Gln466, Trp467, Arg468, Trp469, Arg470, Val471, Phe472, Asp473, Ser475, Ile476, Glu484, Ser487, Leu488, Lys491, Tyr492	6.2
7	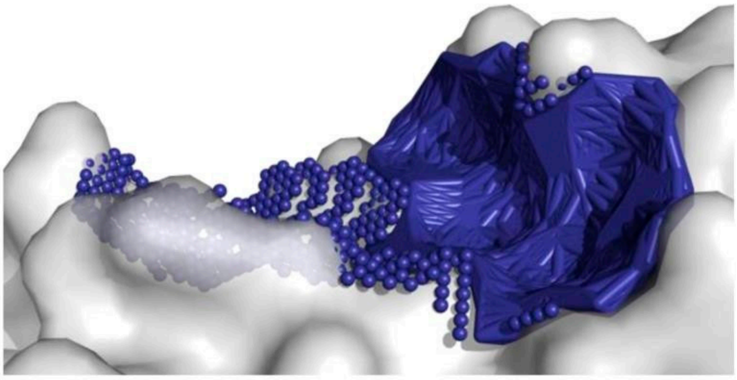<br>The binding pocket is formed by the following amino acid residues: Asn109, Thr110, Thr111, Ile112, Lys113, Ile115, Tyr360, Asn361, Gly362, Lys363, Tyr394, Lys395, Asp396, Leu397, Pro398, Val399, Leu401, Arg402, Ala42, Ala44, Ser45, Lys46, Phe47, Val48, Glu49, Glu50, Tyr51, Asp52,Arg53, Thr54, Gln56, Asn60	-3.5
8	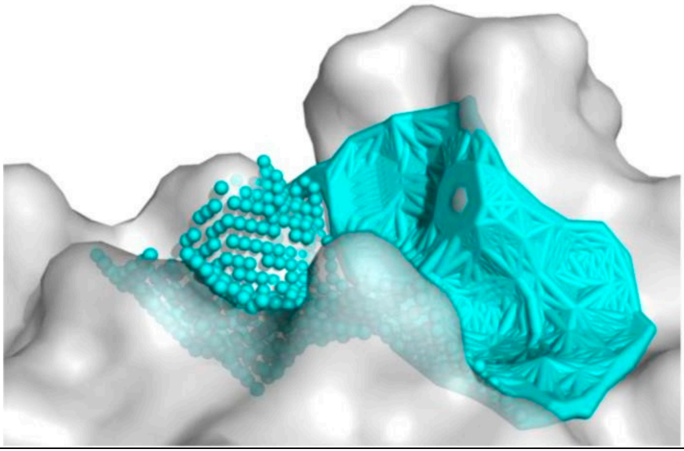<br>The binding pocket is formed by the following amino acid residues: Thr311, Pro312, Arg313, Arg314, Phe316, Phe335, Trp336, Asn337, Lys338, Ser339, Met340, Leu341, Glu342, Lys343, Pro344, Thr345, Asp346, Gly347, Arg348, Val350, Arg366, Ile367, Lys368, Gln369, Thr371, Glu64, Trp67, Asn68, Tyr69, Thr71, Asn72	-0.4

9	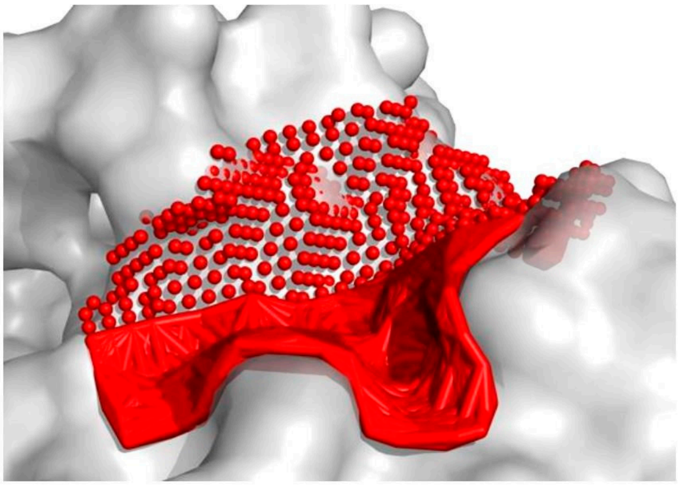	-0.6
	The binding pocket is formed by the following aminoacid residues: Tyr250, Arg253, Ala254,His256, Arg257, His258, Tyr287, Asp288, Leu289, Val290, Val291, Pro292, Phe293, Pro294, Ser295, Glu441, Ile444, Pro598, Asp601, Trp602, Leu603, Arg604, Thr605, Glu606, Asn607,Leu609, His610, Glu612	
10	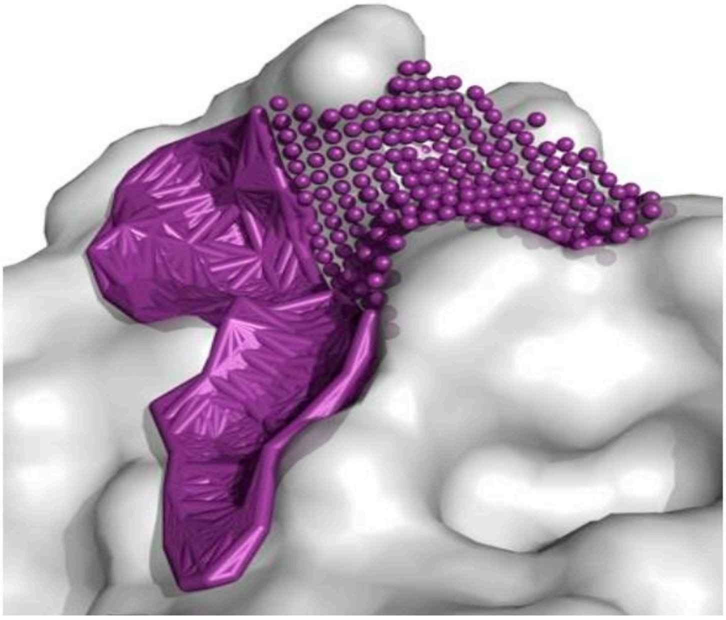	-2.9
	The binding pocket is formed by the following amino acid residues: Gln238, Glu239, Leu240, Gln241, Pro242, Leu243, Leu245, Asn246, Ala249, Lys597, Leu600, Asp601, Trp602, Leu603, Arg604, Thr605, Glu606, Asn607, Glu608, Leu609, His610, Gly611, Glu612, Lys613, Leu614, Gly615	

Table S3. The best 10 identified binding regions on the molecular surface of 2QT9 *receptor.* **For each binding site,the amino acid residues that comprise it have been specified**

No	Results	Free energy values ΔGbinding [kcal/mol]
1	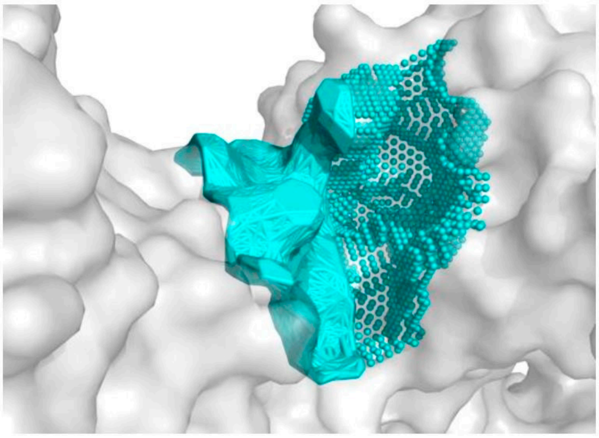<br>The binding pocket is formed by the following amino acid residues: Asn119, Tyr120, Val121, Lys122, Gln123, Trp124, Arg125, Ser127, Tyr128, Thr129, Ala130, Ser131, Asn150, Asn151, Thr152, Gln153, Val167, Trp168, Asn169, Asn170, Asp171, Gly189, Lys190, Glu191, Asp192, Ile193, Ile194, Tyr195, Ile198, Thr199, Asp200, Trp201, Val202, Glu204, Tyr211, Asp230, Thr231,Val233, Pro234, Leu235, Ile236, Glu237, Tyr238, Ser239, Phe240, Tyr241, Ser242, Asp243, Leu246, Tyr248, Pro249, Lys250, Thr251, Val252, Arg253, Val254, Pro255, Tyr256, Thr706, Ala707, Asp708, Asp709, Asp737, Glu738, Asp739, His740	-9.1
2	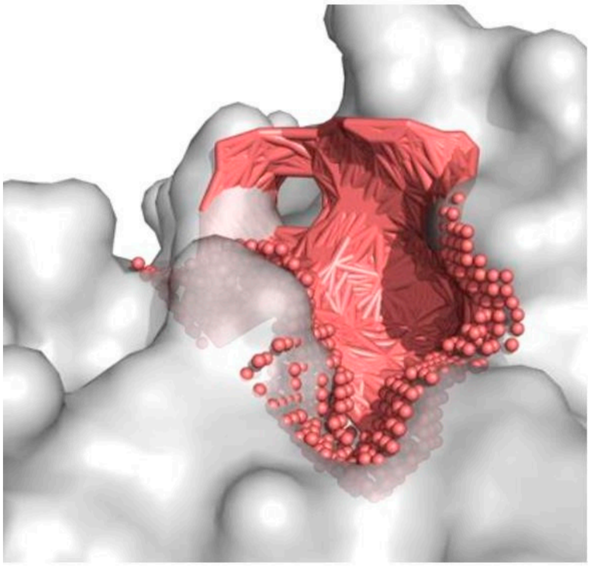<br>The binding pocket is formed by the following amino acid residues: Glu379, Gly380, Tyr381, Gly400, Thr401, Trp402, Glu403, Asn420, Glu421, Tyr422, Lys423, Gly424, Met425, Pro426, Gly427, Phe516, Ile517, Ile518, Leu519, Asn520, Glu521, Thr522, Lys523, Phe524, Trp525, Arg581, Gly584, Tyr585, Gln586, Gly587, Asp588, Lys589, Ile590, Met591, Ala593	-5.5

3	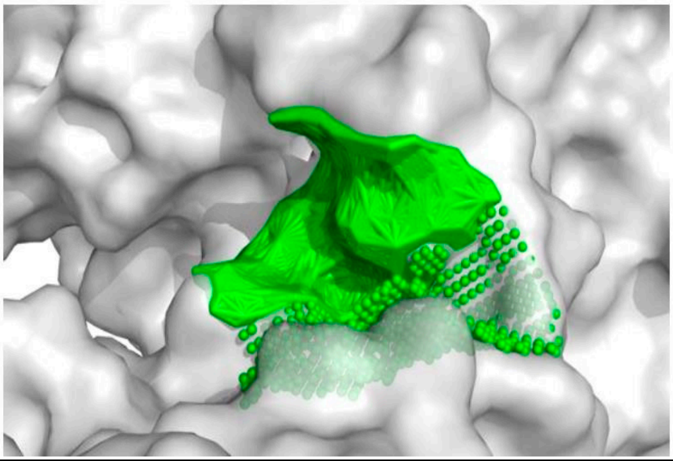<br>The binding pocket is formed by the following amino acid residues: Thr44, Leu45, Thr46, Asp47,Tyr48, Leu49, Lys50, Tyr566, Glu693, Phe695, Lys696, Gln697, Val698, Leu723, Val724, Asp725, Val726, Gly727, Val728, Asp729, Phe730, Gln731, Ser744, Ser745, Thr746, Ala747, His748, Gln749, His750, Ile751, Tyr752, Thr753, His754, Ser756, His757	-5.4
4	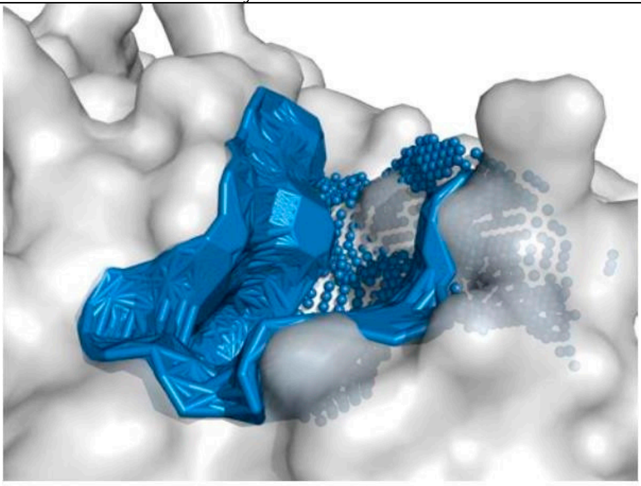<br>The binding pocket is formed by the following amino acid residues: Thr350, Thr351, Ser376,Asn377, Glu378, Glu379, Gly380, Tyr381, Gly400, Thr401, Trp402, Glu403, Asn420, Glu421,Tyr422, Lys423, Gly424, Met425, Pro426, Gly427, Phe516, Ile517, Ile518, Leu519, Asn520, Glu521, Thr522, Lys523, Phe524, Trp525, Asp579, Gly580, Arg581, Gly582, Gly584, Tyr585, Gln586, Gly587, Asp588, Lys589, Ile590, Met591, His592, Ala593, Ile594, Phe601, Asp605	-7.7
5	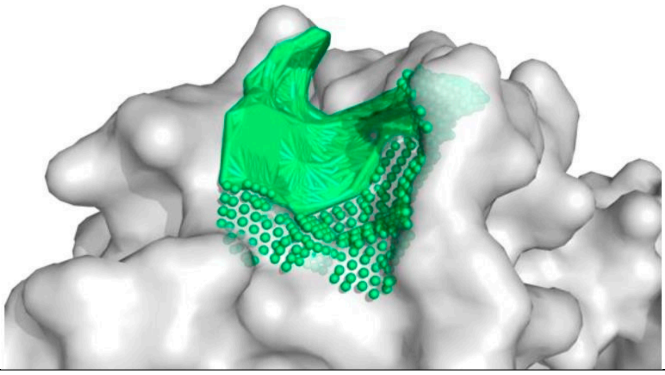<br>The binding pocket is formed by the following amino acid residues: His100, Ile102, Leu116, Asp133, Ile134, Tyr135, Leu137, Lys139, Arg140, Gln141, Leu142, Ile143, Thr144, Phe89, Leu90, Glu91, Asn92, Ser93, Thr94, Phe95, Asp96, Glu97, Phe98, Gly99	-5.4

6	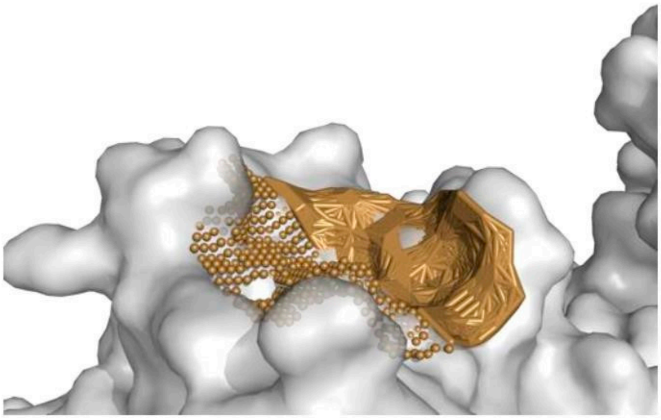	-5.2
	The binding pocket is formed by the following amino acid residues: Thr42, Gln508, Pro531, Pro532, His533, Phe534, Asp535, Ser537, Lys538, Lys539, Tyr540, Pro541, Tyr566, Ala568, Ser569, Thr570, Glu571, Asn572, Ile573, Asp620, Arg623, Lys760, Phe763, Ser764, Leu765, Pro766	
7	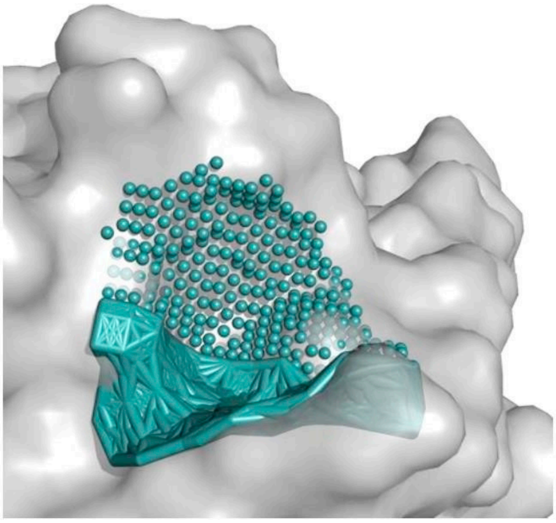	-3.7
	The binding pocket is formed by the following amino acid residues: His100, Leu116, Asp133, Tyr135, Lys139, Arg140, Gln141, Leu142, Ile143, Thr144, Arg147, Ser93, Thr94, Phe95, Asp96, Glu97, Phe98, Gly99	
8	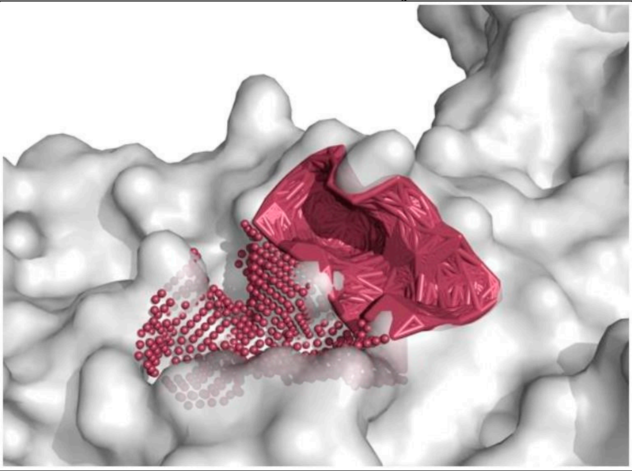	0.7
	The binding pocket is formed by the following amino acid residues: Ile193, Ile194, Tyr203, Gln227, Phe228, Asn229, Asp230, Thr231, Glu232, Val233, Gly260, Ala261, Val262, Asn263, Pro264, Thr265, Val266, Lys267, Phe268, Gln286, Ile287, Thr288, Ala289, Met293, Leu294, Ile295, Gly296, Asp297, His298, Tyr299, Trp315, Arg318	

9	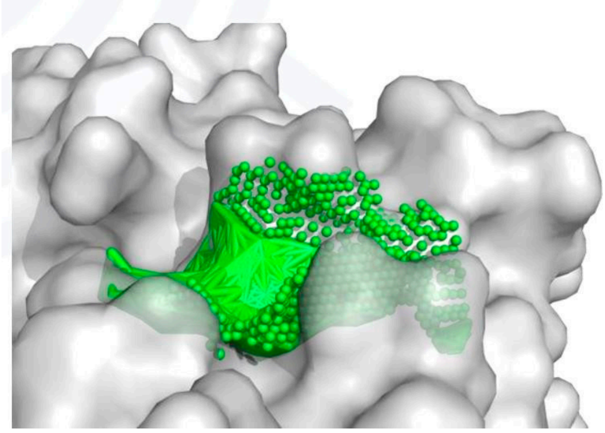	-1.1
	The binding pocket is formed by the following amino acid residues: Ala409, Leu410, Thr411,Ser412, Asp413, Tyr414, Tyr416, Lys433, Gln435, Thr443, Leu445, Phe461, Ser462, Lys463,<br>Glu464, Ala465, Lys466, Tyr467, Ser484, Ser485, Val486, Asn487, Asp488, Asp65, His66	
10	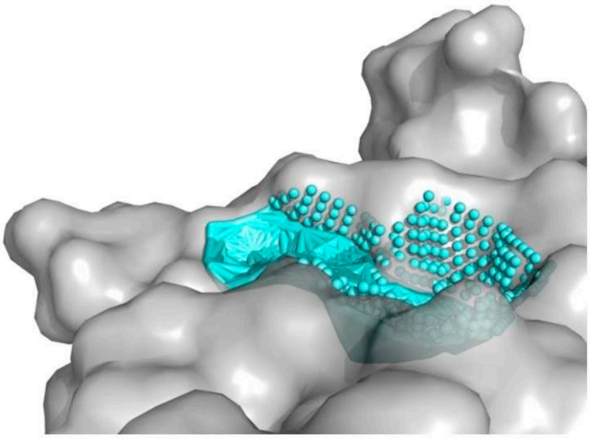	12.1
	The binding pocket is formed by the following amino acid residues: Asp110, Gln112, Ser158, Pro159, Val160, Gly161, His162, Lys163, Leu164, Lys175, Ile176, Glu177, Tyr183, Trp216, Ser217,Pro218, Asn219, Gly220, Thr221, Asn272, Thr273, Asp274, Ser275, Leu276, Ser277	
